# High-efficiency reprogramming of fibroblasts into cardiomyocytes requires suppression of pro-fibrotic signalling

**DOI:** 10.1038/ncomms9243

**Published:** 2015-09-10

**Authors:** Yuanbiao Zhao, Pilar Londono, Yingqiong Cao, Emily J. Sharpe, Catherine Proenza, Rebecca O'Rourke, Kenneth L. Jones, Mark Y. Jeong, Lori A. Walker, Peter M. Buttrick, Timothy A. McKinsey, Kunhua Song

**Affiliations:** 1 Division of Cardiology, Department of Medicine, University of Colorado School of Medicine, 12700 E. 19th Avenue, B139, Aurora, Colorado 80045, USA; 2 Department of Physiology and Biophysics, University of Colorado School of Medicine, 12800 E. 19th Avenue, Aurora, Colorado 80045, USA; 3 Department of Biochemistry and Molecular Genetics, University of Colorado School of Medicine, 12801 E. 17th Avenue, Aurora, Colorado 80045, USA; 4 Charles C. Gates Center for Regenerative Medicine and Stem Cell Biology, University of Colorado School of Medicine, 12700 E. 19th Avenue, B139, Aurora, Colorado 80045, USA

## Abstract

Direct reprogramming of fibroblasts into cardiomyocytes by forced expression of cardiomyogenic factors, GMT (GATA4, Mef2C, Tbx5) or GHMT (GATA4, Hand2, Mef2C, Tbx5), has recently been demonstrated, suggesting a novel therapeutic strategy for cardiac repair. However, current approaches are inefficient. Here we demonstrate that pro-fibrotic signalling potently antagonizes cardiac reprogramming. Remarkably, inhibition of pro-fibrotic signalling using small molecules that target the transforming growth factor-β or Rho-associated kinase pathways converts embryonic fibroblasts into functional cardiomyocyte-like cells, with the efficiency up to 60%. Conversely, overactivation of these pro-fibrotic signalling networks attenuates cardiac reprogramming. Furthermore, inhibition of pro-fibrotic signalling dramatically enhances the kinetics of cardiac reprogramming, with spontaneously contracting cardiomyocytes emerging in less than 2 weeks, as opposed to 4 weeks with GHMT alone. These findings provide new insights into the molecular mechanisms underlying cardiac conversion of fibroblasts and would enhance efforts to generate cardiomyocytes for clinical applications.

## Abstract (web summary)

Direct reprogramming of cardiac fibroblasts into cardiomyocytes is an attractive strategy for heart regeneration, but it is hampered by the low efficiency of the process. Here the authors show that mouse fibroblasts can be reprogrammed with high efficiency into functional cardiomyocytes when pro-fibrotic signaling is inhibited.

## Graphical abstract

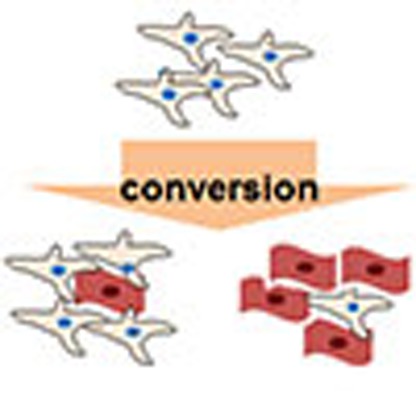

Heart disease is often caused by the loss or dysfunction of cardiomyocytes^1^. The mammalian heart is composed of ∼30% cardiomyocytes, which have limited capacity to regenerate^2^,^3^ and ∼60% endothelial cells and cardiac fibroblasts (CFs)^4^. In injured hearts, CFs are activated by pro-fibrotic signalling such as transforming growth factor-β (TGF-β) and Rho-associated kinase (ROCK) pathways, leading to high-level expression of smooth muscle α-actin (αSMA) and extracellular matrix (ECM) proteins, and culminating in pathological fibrosis^5^,^6^,^7^. Fibroblasts demonstrate plasticity and can be reprogrammed into skeletal muscle cells, induced pluripotent stem (iPS) cells, neurons and hepatocytes^8^,^9^,^10^,^11^. Recently, three transcription factors, GATA4, MEF2C and Tbx5 (GMT), were shown to reprogramme mouse fibroblasts into cardiomyocyte-like cells (iCMs), albeit at low efficiency^12^. Adding Hand2 in GMT (GHMT) enhanced reprogramming efficiency^13^,^14^. Interestingly, forced expression of GMT or GHMT in the myocardium following myocardial infarction (MI) blunted cardiac dysfunction and diminished myocardial remodelling in mice and rats^13^,^15^,^16^,^17^.

Intense investigation has focused on enhancing cardiomyogenic reprogramming, since GMT-mediated reprogramming is slow and inefficient^18^. For example, GMT plus a microRNA, miR-133a, was shown to induce ∼16 beating cells (∼4 beating cells cm^−2^) from mouse embryonic fibroblasts (MEFs) in a well of a 12-well plate after 30 days^19^. Stoichiometry expression of G, M and T protein affects the efficiency of GMT-mediated cardiac reprogramming^20^. Fusion of the MyoD transactivation domain to MEF2C with GHT (MM_3_-GHT) enhanced conversion efficiency^21^. When MEFs transduced with MM_3_-GHT were treated with GSK126, an inhibitor of EZH2 methyltransferase or UNC0638, a potent inhibitor of G9a histone methyltransferase, ∼20% of MEFs spontaneously contracted on day 14 post treatment^22^. In another study, Nkx2–5 was added to GHMT, and MEFs were subsequently treated with SB431542, an inhibitor of the TGF-β type I receptor ALK5 (Tgfbr1), and after 14 days of treatment, ∼16% of these cells displayed calcium transients^23^. Thus, despite extensive efforts, the efficiency of direct reprogramming of embryonic fibroblasts (MEFs) into cardiomyocytes is yet to exceed 20%. More disappointingly, the efficiency of direct reprogramming of adult fibroblasts, including adult CFs (ACFs) and tail-tip fibroblasts (ATTFs), into beating cardiomyocytes is less than 0.1% (refs 13, 19, 20, 21, 22, 23). The inefficiency of direct reprogramming of fibroblasts, especially adult fibroblasts, into functional cardiomyocytes has led many in the field to question the clinical translatability of this method.

Here we demonstrate that pro-fibrotic events are concomitantly activated during GHMT-mediated cardiac reprogramming, leading to inhibiting conversion of fibroblasts into beating cardiomyocytes. Suppression of pro-fibrotic signalling using small molecules that target pro-fibrotic networks provides a remarkably efficient and rapid approach to reprogramming. These findings would facilitate efforts to employ therapeutic cardiac reprogramming and also provide significant and novel molecular insights into the mechanisms underlying reprogramming of fibroblasts into functional cardiomyocytes.

## Results

### Dynamics of pro-fibrotic events during reprogramming

After expression of GHMT, the fraction of cells positive for cardiac troponin T (cTnT), a cardiomyocyte marker, increased with time and reached ∼37% on day 6 (Supplementary Fig. 1a,b). After 1 month, we observed ∼200 (*n*=6) beating cells cm^−2^, which is less than 5% of total cells in dishes (Supplementary Movie 1). GHMT retroviruses infected more than 80% of MEFs (Supplementary Fig. 1c), but less than 5% of infected cells were reprogrammed into beating cardiomyocytes, suggesting endogenous barriers to cardiac reprogramming.

**Figure 1:**
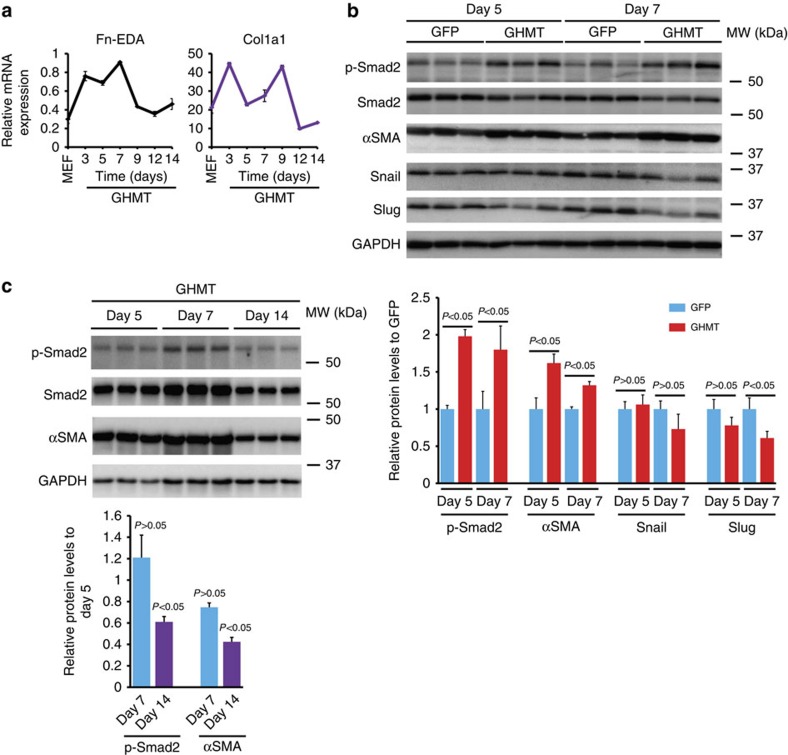
Dynamic changes of pro-fibrotic signalling in reprogramming cells. (**a**) Messenger RNA expression of pro-fibrotic markers, Fn-EDA and Col1a1. Samples were harvested from three independent experiments and measured in duplicate. Data are presented as mean±s.d. Gene expression was normalized to GAPDH. GHMT, GATA4, Hand2, Mef2C, Tbx5. (**b**) Immunoblot (upper) and quantification (lower) of phospho-Smad2 (p-Smad2), αSMA, Snail and Slug in GFP- or GHMT-infected MEFs on days 5 and 7. Data are presented as mean±s.d. *P*>0.05; *P*<0.05 by Student's *t*-test. *N*=3 per group. kDa, kilodaltons; MW, molecular weight. (**c**) Immunoblot (upper) and quantification (lower) of p-Smad2 and αSMA expression in GHMT-infected MEFs on days 5, 7 and 14. GAPDH serves as a loading control. *P* values by Student's *t*-test for comparisons of indicated protein levels between day 7, or day 14 and day 5 are shown. Data are presented as mean±s.d. *N*=3 per group.

To search for endogenous barriers, we performed RNA sequencing (RNA-Seq) to define genes regulated by GHMT overexpression on day 7. We performed gene ontology (GO) enrichment analysis on genes that were upregulated in GHMT-infected cultures. Cardiomyocyte GO terms were enriched among genes upregulated in GHMT-infected cultures (Table 1). Surprisingly, pro-fibrotic/ECM ontology terms^24^ were also significantly enriched among genes upregulated in GHMT-infected cultures on day 7 (Table 1). To confirm RNA-Seq data, we examined fibrotic markers in GHMT-infected MEFs using quantitative RT-PCR (qPCR) or western blot. Expression of ECM genes such as *Fn-EDA* and *Collagen I (Col1a1)* was upregulated after GHMT infection, but downregulated 12 days post infection (Fig. 1a). Immunoblotting also revealed dynamic changes of αSMA expression (Fig. 1b,c and Supplementary Fig. 2). During the first week of induction, pro-fibrotic gene expression in GHMT-infected cultures was higher than that in GFP-infected cultures (Supplementary Fig. 3a). However, by day 14, expression of fibrotic genes in GHMT-infected cultures was ∼50% of that in GFP-infected cultures (Supplementary Fig. 3a). These data suggest that genes involved in fibrotic events are upregulated in GHMT-infected cells during the first week post infection, and expression of these genes wanes after 12 days post-GHMT infection.

TGF-β signalling is an important pathway controlling fibrotic events^5^,^6^,^7^. TGF-β family cytokines, such as TGF-β1/2, bind to type I and type II TGF-β receptors (Tgfbr1 and Tgfbr2), and activate target gene expression by phosphorylating Smad transcription factors, Smad2 and Smad3 (ref. 25). To address whether increasing ECM expression during the early stages of reprogramming correlates with the activation of TGF-β signalling, we analysed the active form of Smad2 with a phospho-specific antibody. Compared with GFP-infected fibroblasts, higher levels of phospho-Smad2 were detected in GHMT-infected cultures on day 5 and day 7 (Fig. 1b and Supplementary Fig. 2). However, phospho-Smad2 and αSMA in GHMT-MEFs were downregulated on day 14, compared with days 5 and 7 (Fig. 1c and Supplementary Fig. 2). In addition, expression of TGF-β signalling components, Tgfb2 and Tgfbr1, was significantly upregulated in GHMT cultures on days, 3, 5 and 7 (Supplementary Fig. 3b). In addition to pro-fibrotic genes, TGF-β signalling regulates downstream targets such as epithelial-to-mesenchymal transition markers, Snail and Slug transcription factors^26^. Overexpression of GHMT downregulated Snail and Slug by day 7, at both the protein and mRNA levels (Fig. 1b, Supplementary Figs 2 and 3c). Taken together, these data suggest that TGF-β signalling and ECM expression are activated in GHMT-infected fibroblasts during the early stages, but are downregulated during later stages of reprogramming. We therefore hypothesized that activation of ECM expression by pro-fibrotic signalling in GHMT-infected fibroblasts suppresses conversion of the cells into cardiomyocytes.

### H3K4me2 marks the cluster of *miR-1-2* and *miR-133a-1*

In addition to searching for potential barriers to GHMT-mediated reprogramming, we attempted to look for enhancers of the process. Enhancement of reprogramming of fibroblasts into iPS cells has been facilitated by genome-wide studies^27^,^28^. To gain insights into global genome changes, we performed chromatin immunoprecipitation followed by deep sequencing (ChIP-Seq) to mark H3K4 dimethylation (H3K4me2) in GHMT-infected MEFs. H3K4me2 marks both promoter and enhancer regions of active genes^29^.

H3K4me2 peaks shifted from a fibroblast towards a cardiomyocyte state post expression of GHMT (Supplementary Fig. 4a). On day 7, 47% of a total of 16,296 H3K4me2 peaks identified in GHMT-expressing cultures were shared with peaks from primary cardiomyocytes. These shifts indicate cardiomyocyte identity caused by GHMT and suggest that expression of GHMT at early stages promotes chromatin changes required for cardiac reprogramming.

It has been shown that muscle-specific microRNAs, miR-1 and miR-133, are critical regulators of skeletal and cardiac muscle cell differentiation^30^,^31^,^32^. H3K4me2 levels at the *miR-1-2/miR-133a-1* locus increased 3 days post-GHMT infection and increased in magnitude with time (Supplementary Fig. 4b). Enhancers ∼2 kb upstream of the *miR-1-2/133a-1* cluster control expression of these two microRNAs in muscle cells^32^,^33^,^34^. Thus, H3K4me2 peaks identified in GHMT-infected fibroblasts mark promoters and enhancers of the *miR-1-2/133a-1* cluster, indicating activation of these two microRNAs. H3K4me2 peaks did not significantly increase at regulatory regions for other muscle-specific microRNAs, including *miR-208* and *miR-499* at such early stages, highlighting the potential importance of miR-1 and miR-133 for initiation of cardiac reprogramming.

### miR-1 and miR-133 enhance cardiac reprogramming

**Figure 2:**
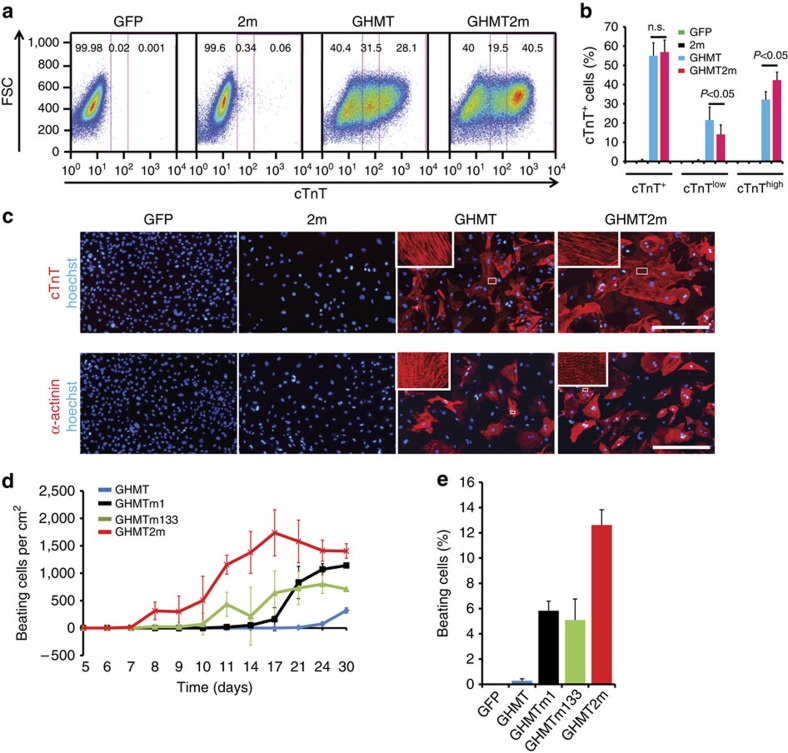
miR-1 and miR-133 enhance GHMT-mediated cardiac reprogramming. (**a**) Flow cytometry analysis for cTnT^+^ cells on day 7 post-viral infection. Numbers embedded in each graph indicate the percentages of cTnT^−^, cTnT^low^ and cTnT^high^ cells shown from left to right. 2m (miR-1+miR-133); GHMT2m (GHMT+miR-1+miR-133); FSC, forward scatter. (**b**) Quantification of cTnT^+^, cTnT^low^ and cTnT^high^ cells shown in **a** (*n*=4 for GFP, *n*=5 for 2m, *n*=11 for GHMT and GHMT2m). Data are presented as mean+s.d. *P*<0.05 by Student's *t*-test. n.s., not significant. (**c**) Representative immunofluorescence images of MEFs stained for cardiomyocyte markers, cTnT (red, upper) and α-actinin (red, lower). MEFs were infected with retroviruses carrying the indicated factors and were cultured for 2 weeks. White boxes are enlarged in insets. Scale bars, 400 μm. (**d**) Time course of spontaneously contracting cells induced by indicated factors (*n*=5 for GHMT, *n*=4 for GHMTm1, *n*=5 for GHMTm133 and *n*=7 for GHMT2m). GHMTm1 (GHMT+miR-1) and GHMTm133 (GHMT+miR-133). Data are presented as mean±s.d. (**e**) Percentages of spontaneously contracting cells induced by indicated factors by 4 weeks. Data are presented as mean+s.d. *N*=4 per group.

To determine whether miR-1 and miR-133 are able to enhance reprogramming, we examined expression of cTnT using flow cytometry 7 days post infection with retroviruses carrying GHMT and/or the microRNAs. GHMT induced expression of cTnT in ∼54% of MEFs (Fig. 2a,b). We divided cTnT^+^ cells into two classes, cTnT^low^ and cTnT^high^. When miR-1 and miR-133 (2m) were delivered alone, less than 1% of cells became positive for cTnT. However, adding 2 m into GHMT (GHMT2m) significantly increased the cTnT^high^ fraction from ∼32 to ∼42% (Fig. 2a,b). Next, we examined effects of 2 m on the assembly of sarcomeres. Immunocytochemistry was performed with antibodies against cTnT and another sarcomeric protein, α-actinin. Two weeks post infection, GHMT produced cells with strong immunostaining of α-actinin and cTnT (Fig. 2c). More positive cells were observed in GHMT2m-infected cultures (Fig. 2c), and expression of cardiomyocyte genes, such as *Actc1*, *Myh6* and *Ryr2*, was also enhanced by 2 m (Supplementary Fig. 5a). These data suggest that miR-1 and miR-133 promote expression of cardiac genes and assembly of sarcomeres.

We next assessed the relative importance of miR-1 and miR-133 for enhancing GHMT-mediated reprogramming. Depletion of either miR-1 or miR-133 from GHMT2m did not significantly reduce the percentage of cTnT^high^ cells in cultures (Supplementary Fig. 5b,c), suggesting that either miR-1 or miR-133 is sufficient to enhance expression of cTnT. Next, we examined spontaneously beating cells. GHMT produced iCMs that started to beat by 3 weeks. GHMT plus miR-1 (GHMTm1) or GHMT plus miR-133 (GHMTm133) produced iCMs that started to beat by 2 weeks and GHMT2m produced iCMs that began beating by 8 days (Fig. 2d and Supplementary Movie 2). More beating iCMs were observed in GHMT2m-infected cultures than with the other conditions (Fig. 2d). By 4 weeks, GHMT, GHMTm1, GHMTm133 and GHMT2m produced ∼200, ∼1,100, ∼700 and ∼1,400 beating iCMs per cm^2^, respectively (Fig. 2d and Supplementary Movie 3). On day 25, GHMT2m induced ∼12% of cultures to spontaneously contract, which is more than beating fractions induced by GHMT, GHMTm1 or GHMTm133 (Fig. 2e). These data suggest that both miR-1 and miR-133 are required for generating maximal beating iCMs in GHMT-infected cultures.

To examine effects of miR-1 and miR-133 on global gene expression, we performed RNA-Seq in GHMT- and GHMT2m-infected MEFs on day 7. Cardiac development ontologies were enriched among genes upregulated in GHMT2m-infected cultures, whereas ECM ontologies were significantly enriched among genes downregulated in GHMT2m-infected cultures (Supplementary Table 1). These data suggest that miR-1 and miR-133 globally enhance cardiac gene expression and inhibit pro-fibrotic gene expression.

### Negative effect of pro-fibrotic signalling on reprogramming

**Figure 3:**
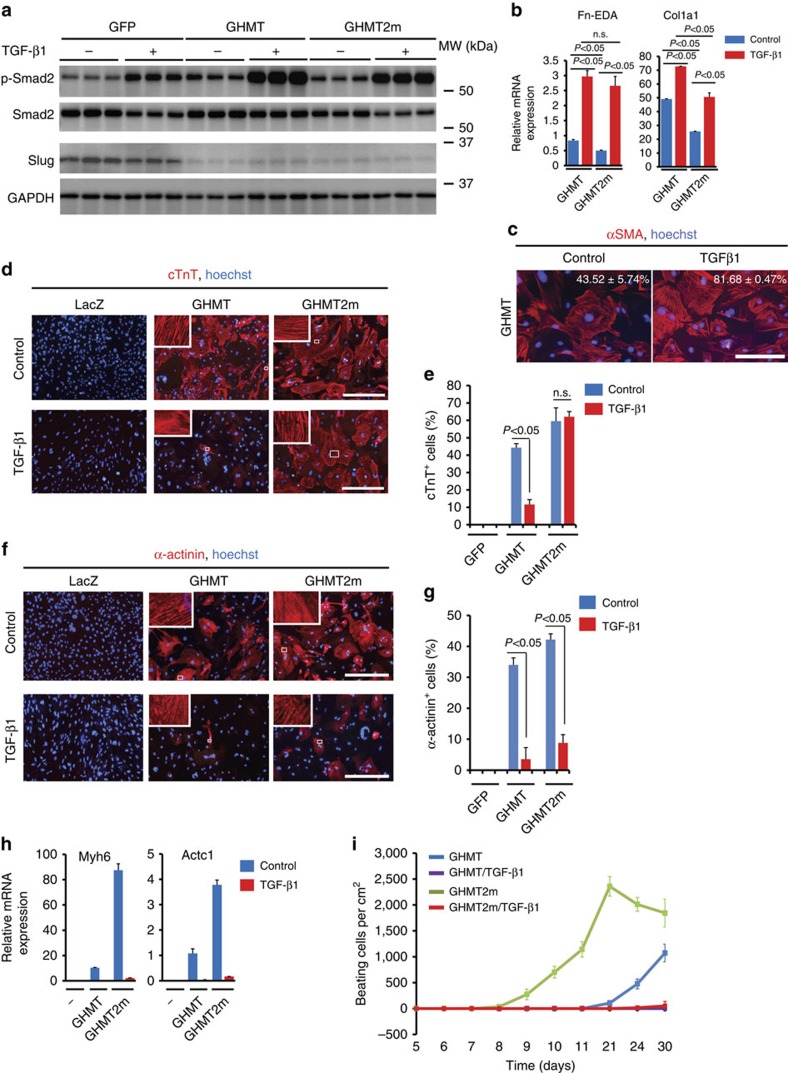
Stimulation of pro-fibrotic signalling by TGF-β1 attenuates cardiac reprogramming. MEFs were infected with indicated retroviral cocktails and then treated with TGF-β1 (10 ng ml^−1^) till the end of experiments. (**a**) Western blot analysis of indicated proteins on day 7. Samples were immunoblotted with antibodies to phospho-Smad2, Smad2, Slug and GAPDH. (**b**) qPCR analysis of the indicated genes on day 7. Gene expression was normalized to GAPDH. *P*<0.05 by Student's *t*-test. *N*=4 per group. n.s., not significant. (**c**) Immunostaining of αSMA^+^ stress fibres in MEFs overexpressing GHMT in the absence or presence of TGF-β1 on day 7. Infected MEFs were treated with TGF-β1 for the 5 days. The number embedded in each plot indicates the percentage of cells with αSMA^+^ stress fibres. Data are presented as mean±s.d. (*n*=3 per group). Scale bar, 200 μm. (**d**–**g**) Representative immunofluorescence images of MEFs stained for cTnT (red; **d**,**e**) and α-actinin (red; **f**,**g**). MEFs were infected with retroviruses carrying the indicated factors and fixed on day 14. White boxes are enlarged in insets. Scale bars, 400 μm. *P*<0.05 by Student's *t*-test. *N*=3 per group. n.s., not significant. Data are presented as mean±s.d. (**e**,**g**). (**h**) qPCR analysis of the indicated cardiac genes in reprogramming cells treated with TGF-β1 or water for 4 days. Non-infected MEFs served as a control. Gene expression was normalized to GAPDH. *N*=3 per group. (**i**) Time course of spontaneously beating cells in the indicated cultures. Data are presented as mean±s.d. *N*=4 per group.

To test the hypothesis that pro-fibrotic signalling serves as a barrier to cardiac reprogramming, we overactivated pro-fibrotic signalling in reprogramming cells. Treatment of fibroblasts with TGF-β1 activates pro-fibrotic signalling cascades^5^,^6^,^7^. TGF-β1 treatment increased phosphorylation of Smad2, expression of *Fn-EDA* and *Col1a1* and formation of αSMA stress fibres in GHMT- or GHMT2m-infected cultures on day 7, compared with non-treated cultures (Fig. 3a–c and Supplementary Fig. 2). These data confirm that TGF-β1 treatment stimulates pro-fibrotic events in GHMT-infected fibroblasts.

Next, we sought to determine whether overactivation of pro-fibrotic signalling suppresses cardiac reprogramming. GHMT induced ∼45 or ∼35% of fibroblasts to be positive for cTnT or α-actinin, respectively, by day 14. Treatment of GHMT-infected cultures with TGF-β1 significantly reduced the cTnT^+^ and α-actinin^+^ populations (Fig. 3d–g). Although treatment with TGF-β1 did not significantly decrease the fraction of cTnT^+^ cells in GHMT2m cultures on day 14 (Fig. 3d,e), expression of other cardiomyocyte markers, including α-actinin, Myh6 and Actc1, was significantly downregulated by treatment with this pro-fibrotic stimulus (Fig. 3f–h). Next, we examined the effect of TGF-β1 treatment on beating iCMs in GHMT- and GHMT2m-infected cultures. GHMT produced ∼1,000 beating iCMs per cm^2^ by 1 month; however, this was reduced by TGF-β1 treatment (Fig. 3i). By 1 month, GHMT2m produced ∼2,000 beating iCMs per cm^2^. TGF-β1 treatment decreased beating iCMs in GHMT2m-infected cultures by 100-fold (Fig. 3i). Taken together, these data demonstrate that overactivation of pro-fibrotic signalling attenuates reprogramming of fibroblasts into beating cardiomyocytes.

### ROCK inhibitors enhance cardiac reprogramming

We next examined whether inhibiting pro-fibrotic events enhances cardiac reprogramming. First, we addressed the impact of blocking ROCK signalling on cardiac reprogramming of fibroblasts. In response to increased mechanical tension, Rho triggers the formation of stress fibres and stimulates pro-fibrotic events via activation of its downstream effector, ROCK. The ROCK inhibitor Y-27632 decreased expression of *Fn-EDA* and *αSMA* but did not change Snail and Slug expression in GFP-, GHMT- and GHMT2m-infected cultures (Supplementary Fig. 2 and Supplementary Fig. 6a–c). These data confirm that Y-27632 suppresses pro-fibrotic genes in reprogramming cells.

**Figure 4:**
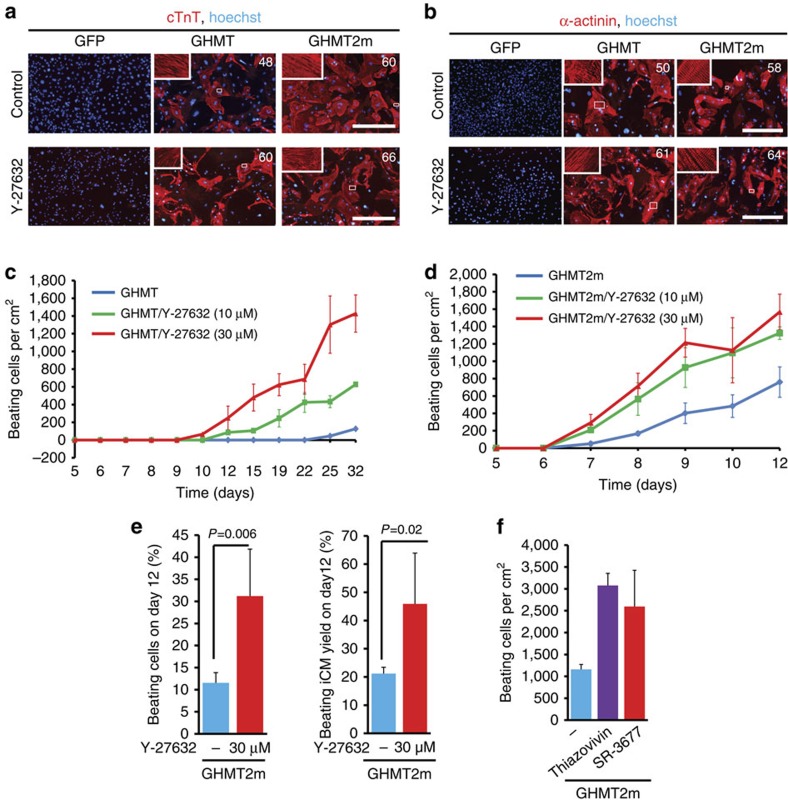
Inhibitors of ROCK signalling enhance cardiac reprogramming. (**a**,**b**) Representative immunofluorescence images of MEFs stained for cTnT (red; **a**) and α-actinin (red; **b**) on day 14. MEFs were infected with retroviruses carrying the indicated factors and treated with Y-27632 (30 μM). The number embedded in each graph represents the average percentage of positive cells. White boxes are enlarged in insets. Scale bars, 400 μm. (**c**) Time course of spontaneously contracting cells in GHMT-infected cultures treated with Y-27632 (10 and 30 μM) or water (*n*=3 per group). Data are presented as mean±s.d. (**d**) Time course of spontaneously contracting cells in GHMT2m-infected cultures treated with Y-27632 (10 and 30 μM) or water (*n*=3 per group). Data are presented as mean±s.d. (**e**) Percentages of spontaneously beating cells (left) and beating iCM yield (right) in GHMT2m cultures treated with or without Y-27632 on day 12. Data are presented as mean+s.d. *P*=0.006; *P*=0.02 by Student's *t*-test. *N*=3 per group. (**f**) Spontaneously beating cells induced by GHMT2m in the presence of the indicated inhibitors of ROCK (*n*=3 per group). GHMT2m MEFs were treated with Thiazovivin (2.5 μM) and SR-3677 (5 μM) for 7 days. Beating cells were counted on day 9. Data are presented as mean+s.d.

Next, we examined the effect of Y-27632 on cardiac reprogramming. Y-27632 treatment enhanced assembly of myocyte sarcomeres (Fig. 4a,b) and cardiomyocyte-specific gene expression (Supplementary Fig. 6d). GHMT induced ∼200 beating iCMs per cm^2^ by 4 weeks. Adding Y-27632 (30 μM) produced contracting iCMs by 12 days and increased the number of beating iCMs by sevenfold by 4 weeks (Fig. 4c). By day 8, GHMT2m produced ∼160 beating iCMs per cm^2^ and GHMT2m plus Y-27632 induced ∼700 beating iCMs per cm^2^ (Fig. 4d). By day 12, we observed ∼1,100 beating cells per cm^2^ and ∼11,000 total cells per cm^2^ in GHMT2m-infected cultures. However, we observed 2,300 beating cells per cm^2^ and 7,000 total cells per cm^2^ in GHMT2m/Y-27632 cultures. Therefore, Y-27632 treatment increased beating cell population from ∼10 to ∼30% in GHMT2m cultures (Fig. 4e). Changes in cell numbers occur during the reprogramming process, which may contribute to the overall efficiency. We calculated the beating iCM yield as the percentage of beating cells in relation to the initial number of plated fibroblasts right before viral infection, as suggested^35^. We observed ∼5,500 fibroblasts right before viral infection. Using this calculation, Y-27632 treatment increased the beating iCM yield from ∼20 to ∼45% in GHMT2m cultures by day 12 (Fig. 4e).

To exclude the possibility of Y-27632 off-target effects, we tested more ROCK inhibitors. Other ROCK inhibitors, including Thiazovivin^36^ and SR-3677 (ref. 37), also enhanced cardiac reprogramming by promoting formation of spontaneously beating iCMs (Fig. 4f). Taken together, these results suggest that small molecules that inhibit pro-fibrotic genes by targeting ROCK signalling significantly enhance reprogramming of MEFs into beating cardiomyocytes.

### Inhibition of TGF-β signalling enhances reprogramming

In addition to the ROCK pathway, the TGF-β pathway plays a critical role in pro-fibrotic gene expression^5^,^6^,^7^. Treatment of MEFs with A83-01, a selective inhibitor of TGF-β signalling^38^, decreased phosphorylation of Smad2, and inhibited expression of *Fn-EDA*, *Col1a1* and *Col3a1*, and formation of αSMA^+^ stress fibers in GHMT- and GHMT2m-infected cultures (Supplementary Figs 2 and 7a–c). We also observed that 2 m significantly decreased expression of *Fn-EDA*, *Col1a1* and *Col3a1* (Supplementary Fig. 7b). Among the four groups of cultures, GHMT, GHMT with A83-01, GHMT2m and GHMT2m with A83-01, the lowest expression of these pro-fibrotic genes was detected in GHMT2m cultures treated with A83-01 (Supplementary Fig. 7b), suggesting that two microRNAs and A83-01 synergistically suppress pro-fibrotic gene expression in reprogramming cells.

**Figure 5:**
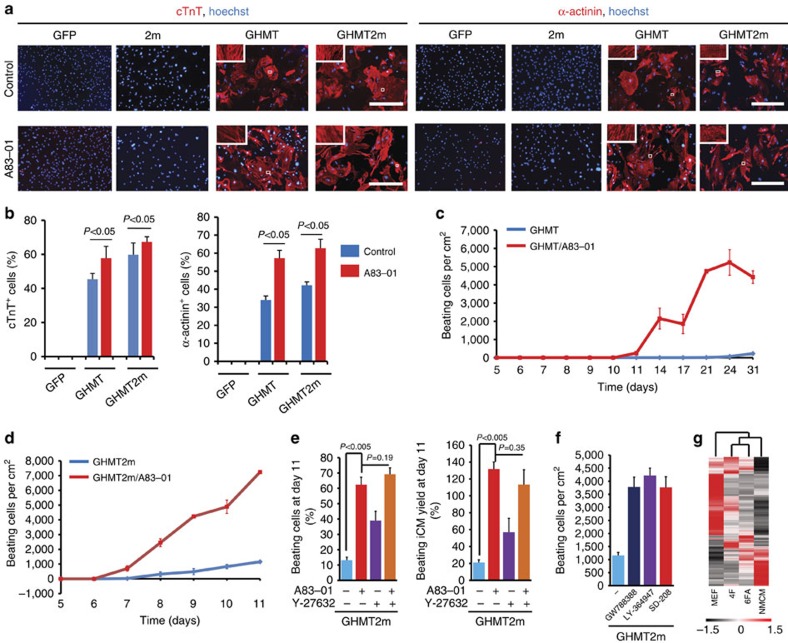
Inhibitors of TGF-β signalling enhance cardiac reprogramming. (**a**) Representative immunofluorescence images of MEFs stained for cTnT (red, left) and α-actinin (red, right) on day 14. White boxes are enlarged in insets. Scale bars, 400 μm. (**b**) Quantification of cTnT^+^ and α-actinin^+^ cells in **a**. Data are presented as mean+s.d. *P*<0.05 by Student's *t*-test. *N*=4 per group. (**c**) Time course of spontaneously contracting cells in GHMT-infected cultures treated with A83-01 (0.5 μM) or dimethylsulphoxide (DMSO; *n*=3 per group). Data are presented as mean±s.d. (**d**) Time course of spontaneously contracting cells in GHMT2m-infected cultures treated with A83-01 or DMSO (*n*=3 per group). Data are presented as mean±s.d. (**e**) Percentages of spontaneously beating cells (left) and beating iCM yield (right) in GHMT2m cultures treated with indicated drugs or DMSO on day 11. Data are presented as mean+s.d. *P*<0.005; *P*=0.19; *P*=0.35 by Student's *t*-test. *N*=3 per group. (**f**) Spontaneously beating cells induced by GHMT2m in the presence of the indicated inhibitors of TGF-β signalling. GHMT2m MEFs were treated with GW788388 (3 μM), LY-364947 (1.5 μM) and SD-208 (1 μM) for 7 days (*n*=3 per group). Beating cells were counted on day 9. Data are presented as mean+s.d. (**g**) Dendrogram cluster and Heat map of RNA-Seq data illustrating differentially expressed 4910 genes at least twofold in fibroblasts (MEF) and primary cardiomyocytes (NMCM). The hierarchical clustering was visualized by using the Pearson correlation similarity and complete linkage algorithm in the Partek Genomics Suite (Partek). RNAs were harvested from MEFs, GHMT (4 F) cultures, GHMT2m+A83-01 (6FA) cultures and NMCM on day 7, followed by deep sequencing.

Given the ability of A83-01 to inhibit pro-fibrotic signalling, we next examined effects of the compound on cardiac reprogramming. A83-01 alone did not induce cTnT^+^ cells, as determined using flow cytometry. In contrast, A83-01 increased cTnT^high^ population from ∼28.1 to ∼30.7% in GHMT-infected cultures and from ∼38.8 to ∼45.6% in GHMT2m-infected cultures on day 7 (Supplementary Fig. 7d,e). More significant increases occurred on day 14. Immunostaining analysis revealed that A83-01 significantly increased the percentages of cTnT^+^ cells in GHMT- and GHMT2m cultures from ∼45%, ∼60% to ∼58%, ∼67%, respectively (Fig. 5a,b). A83-01 also significantly increased the percentages of α-actinin^+^ cells in GHMT- and GHMT2m cultures from ∼34%, ∼42% to ∼57%, ∼64%, respectively (Fig. 5a,b). GHMT2m plus A83-01 induced >60% of fibroblasts to be positive for cTnT or α-actinin (Fig. 5b). A83-01 treatment also enhanced a programme of cardiomyocyte gene expression in reprogramming cells (Supplementary Fig. 7f). The percentage of β-myosin heavy chain (β-MHC) in total MHC (α-MHC+β-MHC) is a critical indicator of cardiomyocyte maturation. There is lower percentage of β-MHC in adult cardiomyocytes than that in neonatal cardiomyocytes (Supplementary Figs 2 and 7g). GHMT2m plus A83-01 generated cardiomyocytes with ∼15% β-MHC by 2 weeks (Supplementary Figs 2 and 7g), suggesting that induced cardiomyocytes are not as mature as adult cardiomyocytes. By 2 weeks, immunostaining analysis showed that expression of myosin light chain-2a (Myl7), an atrial-specific marker, was expressed in ∼70% (*n*=3) of cTnT^+^ cells, whereas the ventricular-specific marker Myl2 was detected in only a few of cells (Supplementary Fig. 7h), suggesting that induced cardiomyocytes are composed of different subtypes of cardiomyocytes.

The dramatic effect of blocking TGF-β signalling was also observed at the level of cell contraction. In the absence of A83-01, beating cells were observed 3 weeks post expression of GHMT and reached ∼200 cells per cm^2^ by 4 weeks (Fig. 5c). Treatment of GHMT-infected cultures with A83-01 led to cell beating by 11 days, which culminated in dramatic and spontaneous contraction of ∼4,400 cells per cm^2^ by 4 weeks (Fig. 5c and Supplementary Movie 4). Even more pronounced changes were observed in GHMT2m-infected cultures. By day 8, GHMT2m reprogrammed a fraction of MEFs into beating cells (∼300 cells per cm^2^) and A83-01 treatment increased the number of beating cells by eightfold (Fig. 5d and Supplementary Movie 5). On day 11, we observed ∼1,000 beating cells per cm^2^ and ∼10,000 total cells per cm^2^ in GHMT2m-infected cultures. However, we observed ∼7,000 beating cells per cm^2^ and ∼11,500 total cells per cm^2^ in GHMT2m/A83-01 cultures. Therefore, A83-01 treatment increased beating cell fraction from ∼10 to ∼60% (Fig. 5e and Supplementary Movie 6). To exclude the effect of changes in cell numbers on the reprogramming efficiency, we calculated the beating iCM yield. The initial number of fibroblast per cm^2^ before viral infection was ∼5,500. Therefore, we obtained the beating iCM yield of ∼120% for GHMT2m plus A83-01 on day 11 (Fig. 5e). This represents the highest efficiency of reprogramming and correlates with the most significant loss of pro-fibrotic gene expression (Supplementary Figs 2 and 7a–c).

Addition of A83-01 and Y-27632 together produced ∼69% spontaneously beating cells in GHMT2m cultures, which is not significantly different from the fraction of beating cells induced by A83-01 alone (Fig. 5e). These data suggest that inhibition of pro-fibrotic events by multiple compounds is not able to further increase the reprogramming efficiency and it will be necessary to manipulate other barriers to further optimize cardiac reprogramming.

To exclude the possibility of A83-01 off-target effects, we examined more inhibitors of TGF-β signalling. Other ALK5 inhibitors, including LY-364947 (ref. 39), SD-208 (ref. 40) and GW788388 (ref. 41) also enhanced cardiac reprogramming by inducing more spontaneously beating cells (Fig. 5f), suggesting that A83-01 enhances cardiac reprogramming through inhibiting TGF-β signalling.

To globally compare the similarity of gene expression in induced cardiomyocytes and primary cardiomyocytes, we performed RNA-Seq on MEFs, neonatal mouse cardiomyocytes (NMCMs), GHMT-infected cultures and GHMT2m-infected cultures treated with dimethylsulphoxide or A83-01 on day 7. Heat map indicates that GHMT2m plus A83-01 not only increases the efficiency but also increases the similarity between iCMs and primary cardiac myocytes, compared with GHMT (Fig. 5g). To gain insights into mechanisms of A83-01 action, we analysed gene expression profiles in GHMT2m cultures treated with or without A83-01 on day 7 by RNA-Seq. The top 10 GO terms that were enriched among genes upregulated in A83-01-treated cultures belong to cardiac development ontologies, whereas the top eight GO terms enriched among genes downregulated in A83-01-treated cultures belong to pro-fibrotic or ECM ontologies (Table 2). These data suggest that small molecules that suppress pro-fibrotic events by targeting TGF-β signalling dramatically decrease pro-fibrotic events and enhance reprogramming of MEFs into beating cardiomyocytes.

### Induced cardiomyocytes display functional properties

**Figure 6:**
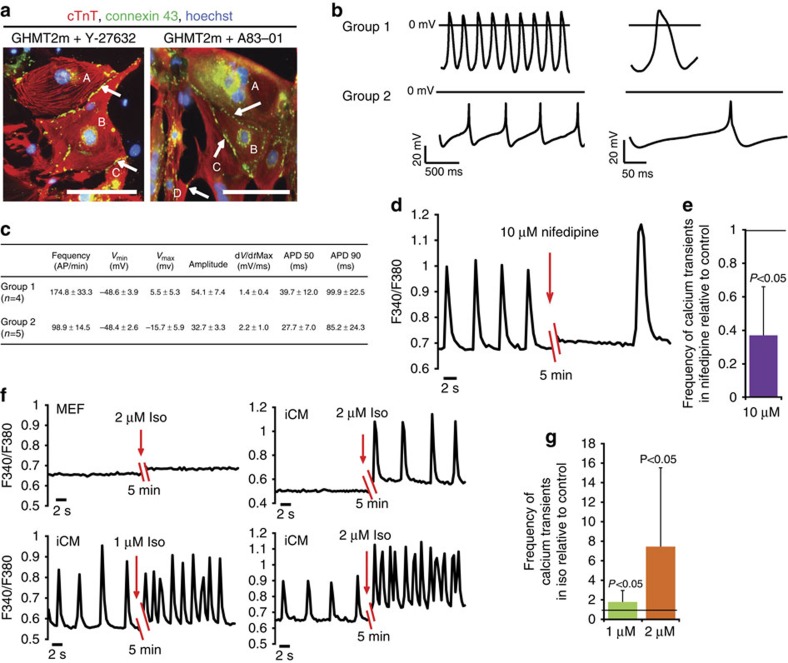
Functional properties of induced cardiomyocytes. (**a**) Immunofluorescence staining of cTnT (red) and cardiac gap junction connexin-43 (green) in GHMT2m-infected cultures treated with Y-27632 or A83-01 on day 14. cTnT^+^ cell A connects to cell B, cell C and cell D via connexin-43 (white arrows). Scale bar, 100 μm. (**b**,**c**) Spontaneous action potentials fired by induced cardiomyocytes in GHMT2m cultures treated with A83-01 on day 9. Representative group 1 or group 2 spontaneous action potentials recorded using the Amphotericin-B-perforated-patch technique are shown in **b**. Single action potentials are expanded on the right. (**c**) Action potential waveform parameters recorded from group 1 and group 2 induced cardiomyocytes including firing rate frequency, the most negative and positive membrane potentials (*V*_min_, *V*_max_), action potential amplitude, maximum upstroke velocity (d*v*/d*t*Max) and the action potential duration at 50 and 90% repolarization (APD50 and APD90, respectively). (**d**) Recorded calcium transients showing effect of 10 μM nifedipine on the calcium-transient frequency of iCMs. F340/F380, ratio of fluorescence intensity at 340 and 380 nm. (**e**) Quantification of calcium-transient frequency from experiments in **d**. Data are presented as mean+s.d. *P*<0.05 versus calcium-transient frequencies before nifedipine treatment by Student's *t*-test. *N*=10. (**f**) Recorded calcium transients showing that treatment with Iso (1 or 2 μM) increases calcium-transient frequency of iCM. F340/F380, ratio of fluorescence intensity at 340 and 380 nm. (**g**) Quantification of calcium-transient frequency from experiments in **f**. Data are presented as mean+s.d. *P*<0.05 versus calcium-transient frequencies before Iso treatment by Student's *t*-test. *N*=21 for the group treated with 1 μM Iso, *n*=14 for the group treated with 2 μM Iso.

A gap junction protein, connexin-43 (Cx43), is responsible for electrical coupling and intercellular communication of cardiomyocytes. Immunostaining revealed that Cx43 was detected along the periphery of iCMs (Fig. 6a), indicating development of gap junction channels.

We next sought to characterize functional properties of these induced cardiomyocytes by electrophysiological analysis. Action potentials (APs) were recorded from single spontaneously beating cells on day 9 of reprogramming. Induced cardiomyocytes fired spontaneous APs (Fig. 6b). Frequency and shapes of APs varied, consistent with a wide range of development or differentiation on day 9 of reprogramming (Fig. 6b,c). APs of induced cardiomyocytes mimic those of fetal (or nodal) cardiomyocytes by showing high rate of spontaneous firing, short AP durations and slow upstroke velocity (Fig. 6c). During cardiac development *in vitro* and *in vivo*, early differentiated cardiomyocytes fire nodal-like APs that specialize into atrial-, ventricular- and nodal-like APs at later differentiation stages^42^,^43^. Therefore, iCMs formed on day 9 appear to mimic early development of cardiomyocytes.

To determine whether iCMs were developing functional excitation–contraction (EC) coupling, we imaged spontaneous calcium transients from Fura-2 AM-loaded iCMs. Spontaneous calcium transients were detected in spontaneously beating cells on days 10–12 (Supplementary Movie 7 and Fig. 6d–g). Addition of nifedipine, a blocker of L-type calcium channels, significantly decreased the frequency of calcium transients, indicating that L-type calcium channels contribute to EC coupling in the iCMs as they do primary cardiomyocytes (Fig. 6d,e). To determine whether iCMs are able to appropriately respond to hormone stimulation, a critical function of normal cardiomyocytes, we measured calcium transients in response to the β-adrenergic agonist isoproterenol (Iso). Addition of 1 or 2 μM Iso significantly increased frequency of cell contraction and spontaneous calcium transients (Fig. 6f,g). In the presence of Iso, some cells that did not beat began to spontaneously contract (Fig. 6f). These data suggest that the development of functional EC coupling machinery and β-adrenergic signalling components in iCMs.

### Reprogramming mediated by adeno-associated viral vectors

Clinical trials using adeno-associated virus (AAV) for gene therapy have shown safety and efficacy. Thus, we sought to determine whether AAV-mediated delivery of reprogramming factors to fibroblasts would lead to conversion to cardiomyocytes. Expression of exogenous GFP delivered by AAV in MEFs was transient, compared with that by retrovirus (Supplementary Fig. 8a). AAV-GHMT2m plus A83-01 induced a small fraction of fibroblasts to be positive for cTnT, α-actinin and to spontaneously contract by day 12 (Supplementary Fig. 8b and Supplementary Movie 8). Although the efficiency of AAV-mediated reprogramming is not comparable to retrovirus-mediated reprogramming, our data suggest that it will be possible to develop an episomal vector system to reprogramme fibroblasts into functional cardiomyocytes, thereby circumventing safety concerns associated with the use of retroviral and lentiviral vectors in humans.

### Reprogramming of adult fibroblasts into beating iCMs

Adult fibroblasts are less amenable to reprogramming than embryonic fibroblasts^44^. The efficiency of direct reprogramming adult fibroblasts into beating cardiomyocytes is less than 0.1%. We therefore examined whether inhibiting pro-fibrotic events could enhance reprogramming of adult fibroblasts, including ACFs and ATTFs, into functional cardiomyocytes.

**Figure 7:**
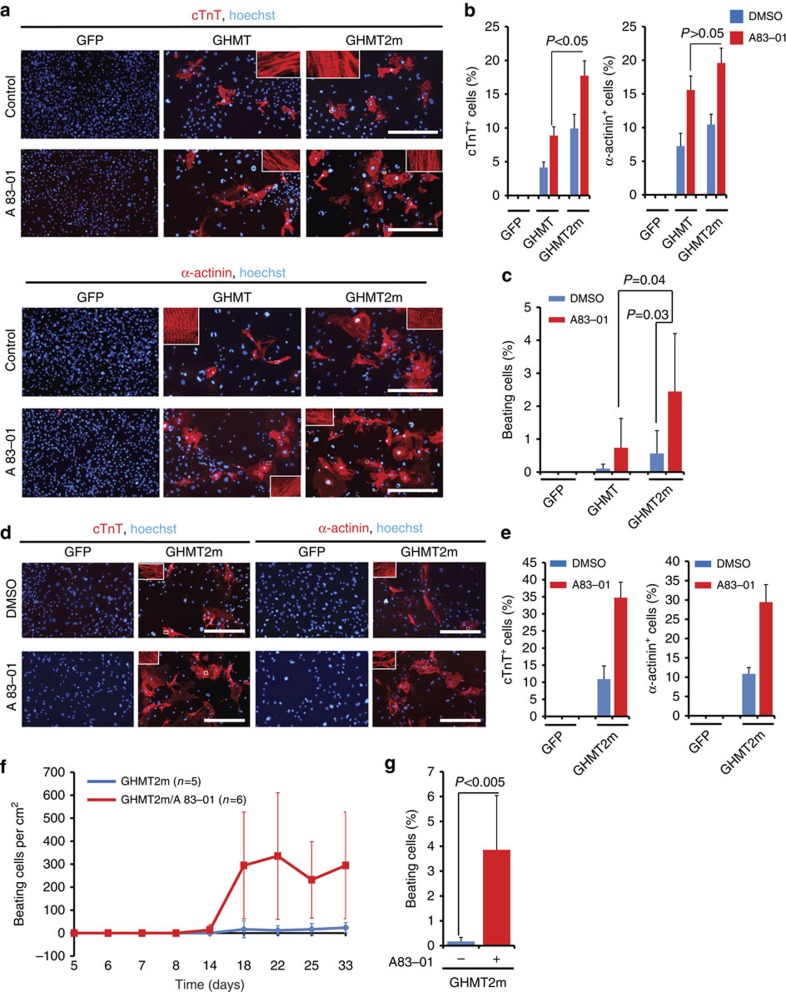
A83-01 enhances reprogramming of adult fibroblasts into beating iCMs. (**a**) Representative immunofluorescence images of ACFs stained for cTnT (red, upper) and α-actinin (red, lower) by 4 weeks. ACFs were isolated from C57BL/6 mice more than 6 months of age. White boxes are enlarged in insets. Scale bars, 400 μm. (**b**) Quantification of cells positive for indicated markers from experiment performed as in **a**. Data are presented as mean+s.d. *P*<0.05; *P*>0.05 by Student's *t*-test. *N*=3 for each group. (**c**) Percentages of spontaneously beating cells in ACFs treated with indicated combinations by 5 weeks. Data are presented as mean+s.d. *P*=0.03; *P*=0.04 by Student's *t*-test. *N*=3 for each group. (**d**) Representative immunofluorescence images of ATTFs stained for cTnT (red, left) and α-actinin (red, right). ATTFs were isolated from C57BL/6 mice more than 6 months of age. ATTFs expressing the indicated factors were treated with A83-01 or DMSO for 2 weeks. Scale bars, 400 μm. (**e**) Quantification of cells positive for indicated markers from experiment performed as in **d**. Data are presented as mean+s.d. *N*=3 per group. (**f**) Time course of spontaneously contracting cells in GHMT2m ATTFs treated with A83-01 (0.5 μM) or DMSO. Data are presented as mean±s.d. (**g**) Percentages of spontaneously beating cells in ATTFs treated with indicated combinations by 5 weeks. Data are presented as mean+s.d. *P*<0.005 by Student's *t*-test. *N*=3 per group.

By 4 weeks, GHMT induced ∼5% of ACFs to be positive for cTnT. Addition of A83-01 significantly increased the cTnT^+^ population to ∼10% (Fig. 7a,b). GHMT2m induced ∼10% of ACFs to be positive for cTnT. A83-01 significantly increased the cTnT^+^ population to ∼18% in GHMT2m ACFs, which is significantly more than the cTnT^+^ fraction induced by GHMT plus A83-01 (Fig. 7a,b). GHMT2m plus A83-01 induced ∼18% of ACFs to be positive for another cardiomyocyte marker α-actinin (Fig. 7b). GHMT plus A83-01 induced ∼0.8% of ACFs to spontaneously contract by 5 weeks; however, GHMT2m plus A83-01 produced ∼2.5% spontaneously beating cells by 5 weeks (Fig. 7c and Supplementary Movie 9). A83-01 also enhanced expression of cardiac genes, *Actc1*, *Myh6* and *Ryr2* (Supplementary Fig. 9a). Therefore, GHMT2m plus A83-01 represents the most optimized combination among the four combinations (GHMT, GHMT plus A83-01, GHMT2m and GHMT2m plus A83-01) to reprogramme ACFs into beating iCMs.

Next, we examined whether A83-01 could enhance GHMT2m-mediated reprogramming of ATTFs into beating cardiomyocytes. GHMT2m reprogrammed a population (∼10%) of ATTFs to be positive for cTnT or α-actinin (Fig. 7d,e). Addition of A83-01 significantly increased the population of cells positive for these markers by ∼20% (Fig. 7d,e). Enhanced cardiomyocyte gene expression was detected in GHMT2m ATTFs treated with A83-01 (Supplementary Fig. 9b). GHMT2m induced ∼40 beating cells per cm^2^ after 1 month. Spontaneously beating cells appeared in A83-01-treated cultures by 3 weeks. By 4 weeks, ∼300 cells per cm^2^ (or ∼4%) spontaneously contracted in A83-01-treated GHMT2m-cultures (Fig. 7f,g and Supplementary Movie 10).

Thus, inhibition of pro-fibrotic signalling also enhances reprogramming of adult cardiac and dermal fibroblasts into functional cardiomyocytes.

## Discussion

**Figure 8:**
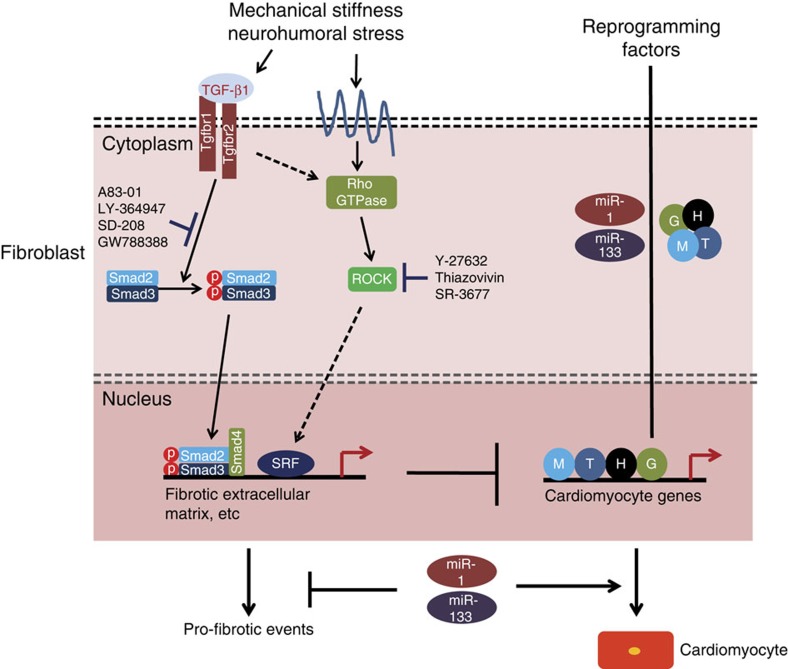
A model for inhibition of cardiomyogenesis by signalling cascades governing fibrotic events. Pro-fibrotic events are governed by TGF-β and/or ROCK signalling. Phosphorylated Smad2 and Smad3 translocate into the nucleus to activate pro-fibrotic genes. Activated ROCK promotes pro-fibrotic events by enhancing actin assembly and activation of pro-fibrotic genes. Pro-fibrotic events serve as barriers to GHMT-mediated cardiac reprogramming. Small molecules that inhibit pro-fibrotic events by inhibiting either TGF-β signalling or RhoA-ROCK signalling are able to enhance reprogramming of fibroblasts into functional cardiomyocytes. miR-1 and miR-133 enhance cardiac reprogramming, at least in part, by attenuating pro-fibrotic gene expression.

Direct conversion of fibroblasts into cardiac muscle represents a promising approach for cardiac regeneration and would benefit patients with ischaemic heart disease^45^,^46^. Here we reveal an effective method for reprogramming fibroblasts into functional cardiomyocytes and demonstrate that pro-fibrotic signalling serves as a major barrier to cardiac lineage reprogramming (Fig. 8). Overactivation of pro-fibrotic signalling by TGF-β1 attenuates cardiac reprogramming. Conversely, inhibition of pro-fibrotic events using small molecules that inhibits either RhoA-ROCK or TGF-β signalling dramatically enhanced the ability of cardiogenic factors to reprogramme mouse fetal and adult fibroblast into beating cardiomyocytes (Fig. 8). These findings suggest potential clinical applications and also provide a novel and facile system to study cardiomyogenesis.

Activation of lineage-specific genes by transcription factors largely depends on chromatin alterations. During differentiation of embryonic stem (ES) cells into cardiomyocytes, promoters of cardiomyocyte genes are gradually marked by H3K4me3 associated with gene activation and expression^47^,^48^. Cardiomyogenesis is regulated by a transcriptional cascade, and the first wave of cardiogenic factors induces the second wave of factors^49^. We speculate that overexpression of GHMT leads to active histone mark(s) at regulatory regions of the key factors promoting cardiomyogenesis in fibroblasts. ChIP-Seq revealed that active histone mark, H3K4me2, significantly increased at the regulatory region of the cluster of *miR-1-2* and *miR-133a-1*. MiR-1 and miR-133 promote cardiac muscle formation by several mechanisms, including inhibiting αSMA expression^30^,^31^,^32^. Our data reveal that miR-1 and miR-133 enhance cardiac reprogramming, at least in part, by attenuating pro-fibrotic gene expression (Supplementary Table 1 and Fig. 8).

Fibrosis is induced by sequential regulation of signalling pathways^50^. Activation of TGF-β signalling occurs early, whereas the delayed activation of the ROCK pathway helps to maintain myofibroblast phenotype. We observed that Y-27632, a ROCK inhibitor, inhibited expression of pro-fibrotic markers less efficiently than A83-01, a TGF-β-signalling inhibitor (Supplementary Figs 6a,c and 7b,c), and this correlated with the generation of induced cardiomyocytes.

TGF-β signalling plays a critical role in diverse biological processes including cell growth, differentiation and development. Here we demonstrate that TGF-β signalling inhibits GHMT-mediated cardiac reprogramming by promoting fibrotic events. Our findings are likely related to the fact that TGF-β signalling inhibits specification and differentiation of cardiomyocytes from mesoderm cells derived from ES cells^51^. Thus, cardiomyogenesis occurring during differentiation of ES cells or GHMT-mediated reprogramming of fibroblasts likely occurs via common mechanisms.

Application of small molecules and iPS-reprogramming factors efficiently reprogrammes mouse fibroblasts into beating cardiomyocytes^52^,^53^. Sequentially adding the JAK inhibitor, JI1 and BMP4, four iPS-reprogramming factors, Oct4, Sox2, Klf4 and c-Myc, induced ∼257 contracting colonies from 100,000 MEFs on day 21 (ref. 52). More recently, in presence of small molecules, SB431542, CHIR99021, parnate and forskolin, Oct4 induced ∼100 contracting colonies from 10,000 MEFs or 50 beating colonies from 10,000 TTFs on day 30 (ref. 53). Here we show that cardiomyocyte lineage factors GHMT and A83-01 induced ∼4,400 beating cells from 5,000 MEFs by week 4. The combination of GHMT2m and A83-01 induced ∼7,000 contracting cells from 5,000 MEFs on day 11, or ∼300 beating cells from 5,000 ACFs/ATTFs by 1 month. In our studies, spontaneously contracting cells did not form colonies, suggesting that GHMT/GHMT2m might induce beating cardiomyocytes from fibroblasts through a path different from pluripotent factor-mediated cardiac reprogramming. Therefore, our studies provide an additional approach to efficiently convert fibroblasts into spontaneously beating cardiomyocytes.

Loss of cardiomyocytes, cardiac hypertrophy and fibrosis are major factors contributing to pathological ventricular remodelling in patients post-MI. Treatment with GW788388 significantly decreased TGF-β activity, cardiac fibrosis and ventricular remodelling with attenuation of systolic dysfunction in rats following MI^41^. The ROCK inhibitor Y-27632 decreased infarct size and remodelling in mice following ischaemia/reperfusion injury^54^. Our findings demonstrate that these small molecules enhance GHMT/GHMT2m-mediated cardiomyocyte conversion by blocking pro-fibrotic signalling (Figs 4 and 5 and Table 2), and highlight the potential of a strategy that combines reprogramming factors with anti-fibrotic compounds as a means of regenerating cardiac tissue post-MI.

## Methods

### Animals

All research involving animals complied with protocols approved by the Institutional Animal Care and Use Committee of the University of Colorado.

### Preparation of MEFs

C57BL/6 pregnant mice at E13 were purchased from Charles River's laboratory. Embryos at E14.5 were harvested and their internal organs and head were removed. The body below the liver was minced to fine pieces. Minced embryos were incubated with 2 ml of 0.25% trypsin/1 mM EDTA, Phenol Red (Gibco) for 40 min at 37 °C with 5% CO_2_. Cells were suspended in 25 ml of growth medium (DMEM/Hi glucose (Hyclone) 10% fetal bovine serum (FBS; Gemini), 1.1% Penicillin–Streptomycin (Gibco) and 1.1% GlutaMAX supplement (Gibco) and then plated on a 15-cm dish. In 24 h, the media were aspirated and new 25 ml of growth medium was added. In 72 h, MEFs were harvested and stored for future use.

### Derivation of adult fibroblasts

Hearts for mouse ACFs or skinned tails for ATTFs from adult C57BL/6 mice were cut into small pieces with ∼1 mm diameter. The biopsies were seeded on a 10-cm culture dish and incubated with 10 ml of growth medium (DMEM/Hi glucose (Hyclone), 10% FBS (Gemini), 1.1% Penicillin–Streptomycin (Gibco) and 1.1% GlutaMAX supplement (Gibco) at 37 °C with 5% CO_2_. Fibroblasts migrated out of the biopsies after 3 days. The media were changed every other day. Once the fibroblasts have reached a 70–80% confluence, the cells were harvested and stored for future use.

### Generation of retroviruses

Twelve micrograms of retroviral plasmid DNA were transfected using FuGENE 6 (Promega) into Platinum E cells (Cell Biolabs) that were plated on a 10-cm tissue culture dish at a density of 3 × 10^6^ cells per dish, 24 h before transfection. Twenty-four hours after transfection, viral medium was harvested and filtered through a 0.45-μm cellulose filter. The viral supernatant was mixed with polybrene (Sigma) to a final concentration of 6 μg ml^−1^.

### Viral infection

Fibroblasts were plated on tissue culture dishes pre-coated with SureCoat (Cellutron) at a density of 40–50 cells mm^−2^ before transduction. Fibroblasts were infected twice with freshly made viral mixture containing polybrene (Sigma) 24 h, 48 h post plating, respectively. Twenty-four hours later, the viral medium was replaced with induction medium composed of DMEM/199 (4:1) (Gibco), 10% FBS (Gemini), 5% horse serum (Gemini), antibiotics (Gibco), 1 × non-essential amino acids (Gibco), 1 × essential amino acids (Gibco), 1 × B-27 (Gibco), 1 × insulin–selenium–transferin (Gibco), 1 × MEM vitamin solution (Gibco) and 1 × sodium pyruvate (Gibco). The medium was changed every 2–3 days until cells were examined.

### Generation of recombinant AAVs

Ten micrograms of total plasmid DNA (1:1:1, pAAV-expression vector: pAAV-DJ: pHelper) were transfected using FuGENE 6 (Promega) into Lenti-X 293T cells (Clontech) growing in DMEM/Hi glucose (Hyclone) supplemented with 10% FBS (Gemini), 1.1% Penicillin–Streptomycin (Gibco) and 1.1% GlutaMAX supplement (Gibco). After 72 h of transfection, cells were harvested with DPBS and 0.5 M EDTA. The cell lysate was treated with Benzonase Nuclease (Sigma) at a final concentration of 50 units per ml for 1 h at 37 °C, and then centrifuged for 15 min at 3,000*g*. Supernatant was collected. AAVs were purified with HiTrap heparin columns (GE Healthcare) and concentrated with an Amicon ultra-4 centrifugal filter unit (Millipore) with a 100,000-molecular-weight cutoff.

### Intracellular staining for flow cytometry

Adherent cells were washed with DPBS (Gibco). Cells were detached from culture dish by treatment with 0.25% Trypsin/EDTA (Gibco) for 3 min at 37 °C. One million cells were washed with 500 μl of 1% BSA in DPBS and fixed with 0.2 ml of BD Cytofix/Cytoperm solution for 20 min on ice. Cells were washed with 1 ml of BD Perm/wash buffer twice. Cells were incubated with 100 μl of blocking buffer containing 5% of goat, donkey serum (Sigma: G9023 and D9663) in Perm/wash buffer for 30 min at room temperature (RT). Cells were incubated with primary antibodies: Mouse Troponin T (cTnT) antibody (Thermo Scientific, ms-295-p, 1:200) or mouse anti-c-Myc antibody (BD Pharmingen, 551102, 1:200) in 100 μl BD Perm/Wash buffer for 1 h at RT. Cells were washed with 1 ml of ice-cold BD Perm/wash buffer twice. Cells were incubated with the secondary antibody, anti-mouse Alexa 647 (Life Technologies, A-31571, 1:200) in 100 μl BD Perm/wash buffer for 45 min at RT. Cells were washed twice with 1 ml cold BD Perm/wash buffer and re-suspended in 300 μl of 1% BSA/DPBS and then analysed using the FACS Caliber (BD Sciences) and FlowJo software.

### Quantitative RT–PCR (qPCR)

Total RNA was extracted with TRizol reagent (Ambion) and the RNeasy Plus Universal Mini Kit (Qiagen). cDNA was synthesized with the Superscript III First-Strand Synthesis System (Invitrogen). qPCR was performed using the SYBR Green PCR Master Mix (Applied Biosystems) or StepOne Real-Time PCR Systems (Applied Biosystems). Ryr2 (Mm00465877_m1) and Myh7 (Mm01319006_g1) are Taqman probes (Life Technologies). The details about primers are in Supplementary Table 2.

### ChIP-Seq and RNA-Seq

Chromatin immunoprecipitation with antibody against H3K4me2 (Abcam, Ab7766) was performed^55^. Briefly, Cells were crosslinked with 1/10 volume of fresh 11% formaldehyde solution (50 mM HEPES-KOH, pH 7.5, 100 mM NaCl, 1 mM EDTA, 0.5 mM EGTA, 11% formaldehyde) at RT for 10 min. One hundred million cells in 3 ml of lysis buffer 3 (10 mM Tris-HCl, pH 8.0, 100 mM NaCl, 1 mM EDTA, 0.5 mM EGTA, 0.1% Na-Deoxycholate, 0.5% N-lauroylsarcosine and protease inhibitors (Roche) were sonicated with a microtip attached to FB-120 sonicator (Fisher) to produce DNA fragments ranging from 100 to 600 bp. Supernatant was harvested for immunoprecipitation. Ten microlitres of supernatant were used as an input control for deep sequencing. Sonicated DNA was incubated with 20 μl magnetic bead (Thermo Scientific, 26162) and 8 μl antibody against H3K4me2 at 4 °C overnight. Complex of bead–antibody–DNA was collected by a MagneSphere Technology Magnetic Separation Stand (Promega, Z5342), and then washed in 1 ml Wash Buffer-RIPA (50 mM HEPES-KOH, pH 7.6, 500 mM LiCl, 1 mM EDTA, 1% NP-40, 0.7% Na-Deoxycholate) three to seven times. The DNA–protein complex was eluted by using 210 μl of elution buffer (50 mM Tris-HCl, pH 8.0, 10 mM EDTA, 1% SDS) and reversed. DNA was purified using the MinElute PCR Purification Kit (Qiagen, 28004). ChIP-seq library preparation and deep sequencing were performed at Genomics and Microarray Core at the University of Colorado-Auschutz Medical Campus. Illumina HiSeq fastq files were processed to trim off 3-prime end low-quality bases, mapped to the mm9 genome with the Genomic Short-read Nucleotide Alignment Program v2012-17-20 (ref. 56) and the resulting SAM files converted to sorted, indexed BAM files with SAMtools v0.1.19-44428 cd (ref. 57). The BAM files were analysed using PhantomPeaks v2.0 (ref. 58). Peaks were called using the Hypergeometric Optimization of Motif EnRichment suite v4.7 (HOMER)^59^ by the findPeaks command using input DNA as the background with the following options: size 1,000, minDist 2,500 and fraglength equal to the DNA fragmentation length determined by PhantomPeaks (ranging from 250 to 297). Differential peaks were determined using the getDifferentialPeaks HOMER command with the default settings, and all peaks were annotated using the HOMER annotatePeaks.pl programme against mm9. Peak shifts were calculated using the HOMER mergePeaks command with the –venn and –prefix options. ChIP-seq peaks were visualized by uploading bedGraph files, generated using the HOMER makeUCSCfile command, into the UCSC genome browser^60^ as custom tracks. ChIP-Seq data have been deposited in Gene Expression Omnibus at NCBI under accession code GSE71392. Total RNA was extracted with TRizol reagent (Ambion) and the RNeasy Plus Universal Mini Kit (Qiagen). RNA-seq library preparation and deep sequencing were performed at Genomics and Microarray Core at the University of Colorado-Auschutz Medical Campus. Data analysis was performed using the Partek Flow software, version 3.0 Copyright; 2014 Partek Inc., St Louis, MO, USA. RNA-Seq data have been deposited in Gene Expression Omnibus at NCBI under accession code GSE71405.

### Recombinant peptide and small molecules

Recombinant peptide and small molecules are as follows: A83-01 (Tocris 2939), Y-27632 (Enzo Life Science ALX-270-333-M005), Recombinant Mouse TGF-β 1 (Novoprotein CA59, R&D Systems 7666-MB-005), GW788388 (Sigma-SML0116-5 mg), LY-364947 (Sigma-L6293-5MG), SD-208 (Sigma-S7071-5MG), Thiazovivin (Sigma-SML1045-5 mg) and SR-3677 (Sigma-SML0774-5 mg).

### Western blot

Cells were washed with ice-cold DPBS (Gibco) twice, and then ice-cold lysis buffer (150 mM NaCl, 50 mM Tris-Cl pH 7.4, 1 mM EDTA, 1% Triton, Complete mini tablet (Roche), 1 mM phenylmethylsulphonyl fluoride freshly added before use) was added to the cells. Twenty micrograms of lysate were loaded for analysis. The primary antibodies include the following: Snail (Cell Signaling, 3879S, 1:1,000), Slug (Cell Signaling, 9585S, 1:1,000), P-Smad2 (Cell Signaling, 3108P, 1:1,000), Smad2 (Cell Signaling, 5339, 1:1,000), αSMA (Santa Cruz, SC-32251, 1:10,000) and GAPDH (Ambion, AM4300). The secondary antibodies include the following: Goat Anti-Mouse IgG (H+L; Southern Biotech, 1031-05, 1:2,000) and Goat Anti-Rabbit IgG (Life Technologies, 65–6120, 1:2,000). For separation of α- and β-myosin heavy chain, samples were run by modified 6% SDS–PAGE (separating acrylamide/*bis*-ratio 1:100; resolving gel buffer pH 9.0; running gel buffer pH 8.2; β-mercaptoethanol 600 μl l^−1^ inner gel buffer). Gels were run overnight at 4 °C and stained with BioSafe Coomassie Blue protein stain. Quantification of band intensities was performed by densitometry of images using the Image J software.

### Immunocytochemistry

Cells were fixed in 2% paraformaldehyde for 15 min on ice and then washed with DPBS (Gibco) three times at room temperature (RT). The cells were permeabilized in 0.5% Triton X-100 (made in PBS) for 20 min at RT. After blocked in DPBS containing 10% FBS for 30 min at RT, cells were incubated with the primary antibody against α-actinin (Sigma A7811, 1:300), Connexin-43 (Sigma, C6219, 1:500), Troponin T (Thermo Scientific MS-295-P, 1:500), α-SMA (Santa Cruz Biotechnology, SC-32251, 1:800), Myl2 (Proteintech, 10906-1-AP, 1:200) or Myl7 (Proteintech, 17283-1-AP, 1:200) in 10% FBS (Gemini) in DPBS for 1 h at RT. Cells were washed with DPBS three times and then incubated with the secondary antibody (Alexa Fluor 555 Molecular Probes A21422, 1:1,000) and Hoechst (Molecular Probes 33342, 1:5,000) for 30 min at RT. Quantification of positive cells for cTnT, α-actinin and αSMA was performed from at least three independent experiments. Twenty to thirty fields with an area of 0.89 mm^2^ were randomly chosen from each experiment. The mean of percentage of positive cells in these 20–30 fields represents the percentage of positive cells in each experiment.

### Counting of beating cells

Cells in 60-mm dishes were examined under an EVOS FL Color Imaging System (Life Technologies) at 25 °C. Beating cells were counted at a field with an area of 0.89 mm^2^. Ten to fifteen fields were randomly chosen for each dish.

### Recording videos of beating cells

Beating cells were visualized on an inverted microscope (Nikon Diaphot 300), and videos were obtained using a CCD (charge-coupled device) camera (Point Grey Research, Flea 2) at 25 °C.

### Recording of spontaneous APs

Fibroblasts were plated on fibronectin-coated glass coverslips (BD BioCoat #354088) followed by viral infection and drug treatment. Fibroblasts were sparsely plated for electrophysiological recordings and isolated cells were selected for patching to minimize AP waveform distortions arising from intercellular electrical coupling. Fragments of glass coverslips plated with iCMs on day 9 were transferred to a recording chamber (200 μl) on the stage of an inverted microscope. Cells were constantly perfused (1–2 ml min^−1^) with normal Tyrode's solution at 35±1 °C (in mM, 140 NaCl, 5.4 KCl, 1.2 KH_2_PO_4_, 5 HEPES, 5.55 glucose, 1 MgCl_2_, 1.8 CaCl_2_; pH-adjusted to 7.4 with NaOH). Spontaneous APs were recorded from spontaneously beating cells in the amphotericin perforated-patch configuration in current–clamp mode (Axopatch 200B, Molecular Devices). The pipette solution contained (in mM) 135 KCl, 0.1 CaCl_2_, 1 MgCl_2_, 5 NaCl, 10 EGTA, 4 Mg-ATP and 10 HEPES, with pH adjusted to 7.2 with KOH and Amphotericin-B (Fisher Scientific, BP928) added at a final concentration of 100 μg ml^−1^. APs were analysed off-line using the ClampFit software (Molecular Devices). AP firing rates for each cell are reported as the average instantaneous firing rate measured during 15–30 s recording windows. AP waveform parameters were calculated for each cell from average waveforms from 5-s recording windows as we have previously described^61^.

### Measurements of calcium transient

For calcium imaging, beating iCMs or MEFs were loaded with 5 μM Fura-2 AM (Life Technologies) together with 0.1% Pluronic F-127 (Life Technologies) in modified Tyrode's solution (140 mM NaCl, 5 mM KCl, 1.8 mM CaCl_2_, 1 mM MgCl_2_, 10 mM glucose and 10 mM HEPES, pH 7.4) containing 0.1% BSA and 1% pyruvate for 30 min at 37 °C while shielded from light. Before imaging, the cells were washed and allowed to de-esterify the Fura-2 AM for 30 min in Tyrode's solution at RT. Ca^2+^ imaging was performed at RT (∼25 °C) using Slidebook 5.5 Ca^2+^ Imaging System (3I Marianas Spinning Disk) with an automated fluorescence microscope and a CCD camera at the Advanced Light Microscopy Core, University of Colorado Anschutz Medical Campus. When it is applied, 1 or 2 μM isoproterenol (Sigma) or 10 μM Nifedipine (Sigma) was applied locally to the cell. Calcium transients in individual spontaneous beating cell were calculated by the ratio of fluorescence intensity at 340 nm to that at 380 nm.

### Statistical analysis

Statistical analysis was performed using the two-sided Student's *t*-test. *P*<0.05 was considered statistically significant.

## Additional information

**How to cite this article:** Zhao, Y. *et al.* High-efficiency reprogramming of fibroblasts into cardiomyocytes requires suppression of pro-fibrotic signalling. *Nat. Commun.* 6:8243 doi: 10.1038/ncomms9243 (2015).

## Supplementary Material

Supplementary Information Supplementary Figures 1-9, Supplementary Tables 1-2 and Supplementary References

Supplementary Movie 1 Spontaneously beating cells in GHMT-MEFs by one month.

Supplementary Movie 2 Spontaneously beating cells in GHMT2m-MEFs at day 11.

Supplementary Movie 3 Spontaneously beating cells in GHMT2m-MEFs by 3 weeks.

Supplementary Movie 4 Spontaneously beating cells in GHMT-MEFs treated with A83-01 by 3 weeks.

Supplementary Movie 5 Spontaneously beating cells in GHMT2m-MEFs treated with A83-01 at day 8.

Supplementary Movie 6 Spontaneously beating cells in GHMT2m-MEFs treated with A83-01 at day 12.

Supplementary Movie 7 Calcium transients in GHMT2m-MEFs with A83-01 at day 10. Cells were incubated with Fura-2 AM. Green fluorescence represents excitation at 340 nm, and red fluorescence represents excitation at 380 nm.

Supplementary Movie 8 A spontaneously beating cell in AAV-GHMT2m-MEFs treated with A83-01 by 12 days.

Supplementary Movie 9 Spontaneously beating cells in GHMT2m-ACFs treated with A83-01 by 4 weeks.

Supplementary Movie 10 Spontaneously beating cells in GHMT2m-ATTFs treated with A83- 01 by 3 weeks.

## Figures and Tables

**Table 1:** GO analysis of genes upregulated by overexpression of GHMT in fibroblasts.

**Genes**	**GO description**	**Enrichment score**	***P-*****value**
Cardiac development	Contractile fibre part	42.03	5.57E−19
	Muscle system process	31.15	2.97E−14
	Z disc	30.84	4.03E−14
	Regulation of heart contraction	25.5	8.45E−12
	Regulation of cardiac muscle contraction	17.43	2.68E−08
	Cardiac muscle contraction	16.23	8.92E−08
	Myofibril assembly	13.07	2.11E−06
	Positive regulation of heart growth	13.39	1.53E−06
	Cardiac conduction	12.72	2.99E−06
	Cell communication involved in cardiac conduction	12.5	3.71E−06
	Cardiac muscle tissue morphogenesis	11.14	1.46E−05
	Cardiac muscle cell action potential	10.82	1.99E−05
	Regulation of heart rate	10.51	2.72E−05
	Ventricular cardiac muscle cell action potential	9.98	4.63E−05
	Regulation of cardiac muscle contraction by calcium ion signalling	9.87	5.16E−05
	Cardiac muscle cell development	9.77	5.70E−05
Fibrotic events	Extracellular region part	33.23	3.69E−15
	Extracellular organelle	26.76	2.40E−12
	Extracellular vesicular exosome	26.76	2.40E−12
	Extracellular membrane-bounded organelle	26.76	2.40E−12
	Basement membrane	21.5	4.60E−10
	Extracellular matrix	19.2	4.57E−09
	Extracellular matrix organization	13.24	1.78E−06
	Extracellular structure organization	13.04	2.17E−06
	Collagen trimer	10.41	3.02E−05

GO, gene ontology; GHMT, GATA4, Hand2, MEF2C and Tbx5; MEF, mouse embryonic fibroblast; RNA-Seq, RNA sequencing.

RNA-Seq was performed to analyse gene expression in MEFs and MEFs infected with GHMT on day 7. GO analysis of 3,517 genes upregulated ≥1.5-folds in GHMT cultures demonstrates that forced expression of GHMT upregulated genes involved in cardiomyogenesis and pro-fibrotic events on day 7. *P* values were computed by using the Partek Flow software (Partek).

**Table 2:** GO analysis categorizes genes that are up- or downregulated at least twofold in GHMT2m cultures treated with A83-01, compared with GHMT2m cultures treated with DMSO on day 7.

**Genes**	**GO description**	**Enrichment score**	***P-*****value**
*GHMT2m cultures treated with A83-01*			
Upregulated			
Cardiac development			
Cardiac muscle development	Contractile fibre part	41.24	1.24E−18
	Z disc	31.31	2.53E−14
	Muscle system process	30.09	8.53E−14
	Muscle contraction	23.93	4.03E−11
	Regulation of heart contraction	23.64	5.44E−11
	Regulation of heart rate	23.23	8.18E−11
	Sarcomere	22.28	2.10E−10
	Sarcoplasmic reticulum	19.96	2.15E−09
	Muscle tissue morphogenesis	19.88	2.33E−09
	Calcium ion binding	19.83	2.43E−09
	Striated muscle contraction	19.24	4.41E−09
	Cardiac muscle tissue morphogenesis	18.99	5.68E−09
Mitochondrion	Mitochondrion	31.2	2.83E−14
	Mitochondrial inner membrane	26.72	2.50E−12
	Mitochondrial membrane	22.25	2.17E−10
	Respiratory chain	21.32	5.51E−10
	Mitochondrial part	20.08	1.91E−09
	Mitochondrial membrane part	19.71	2.76E−09
	Transmembrane transporter activity	19.27	4.29E−09
Downregulated			
Fibrotic events	Extracellular matrix	89.58	1.25E−39
	Extracellular region	87.72	7.98E−39
	Proteinaceous extracellular matrix	74.47	4.56E−33
	Extracellular matrix part	51.09	6.46E−23
	Extracellular region part	45.82	1.27E−20
	Extracellular space	44.41	5.15E−20
	Extracellular matrix organization	41.91	6.29E−19
	Extracellular structure organization	41.72	7.61E−19

DMSO, dimethylsulphoxide; GO, gene ontology; GHMT2m, miR-1 and miR-133 into GHMT; GHMT, GATA4, Hand2, MEF2C and Tbx5.

Nineteen of top twenty-five ontologies enriched among 509 upregulated genes are related to cardiac development. Top eight ontologies enriched among 489 downregulated genes are related to pro-fibrotic events. *P* values were computed by using the Partek Flow software (Partek).
