# Supplementary material for: High-efficiency reprogramming of fibroblasts into cardiomyocytes requires suppression of pro-fibrotic signalling
Source: Nat Commun. 2015 Sep 10;6:8243. doi: 10.1038/ncomms9243 (PMC4579788; doi:10.1038/ncomms9243)
Supplement: Supplementary Information — Supplementary Figures 1-9, Supplementary Tables 1-2 and Supplementary References [file ncomms9243-s1.pdf]

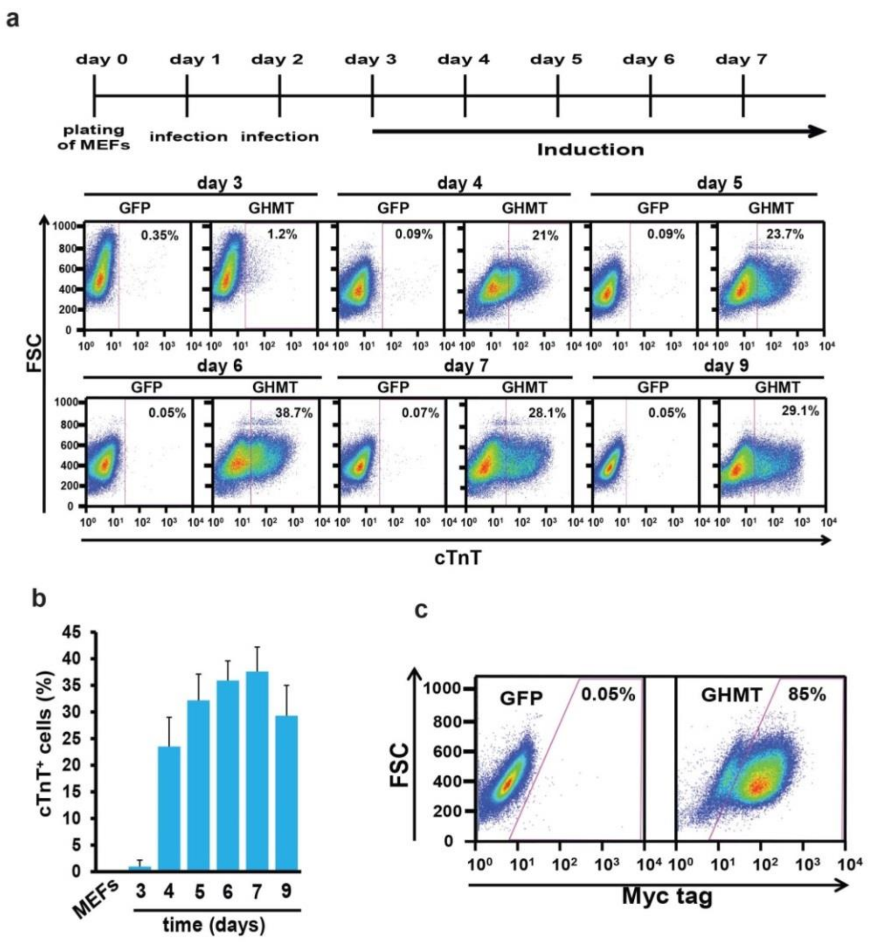

Supplementary Figure 1. Induction of cTnT expression by GHMT. (**a**) Time course of cTnT expression induced by GHMT. Scheme of factor-mediated cardiac reprogramming is shown (upper). MEFs were prepared from C57Bl6 embryos at embryonic day 14.5 (E14.5). The number embedded in each plot indicates percentage of cTnT$^+$ cells (lower). FSC, forward scatter. (**b**) Quantification of cTnT$^+$ cells analyzed by flow cytometry in (**a)** (n = 2). Data are presented as mean + s.d. (**c**) Flow cytometry analysis for expression of reprogramming factors in MEFs. MEFs were infected with retroviruses encoding GFP or GHMT (each factor tagged with Myc) and analyzed at day 6. The percentage of cells positive for Myc expression in each group is shown.

Fig. 1b

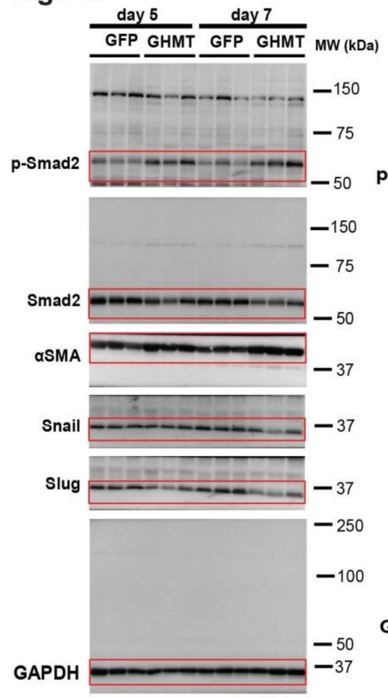

Fig. 1c

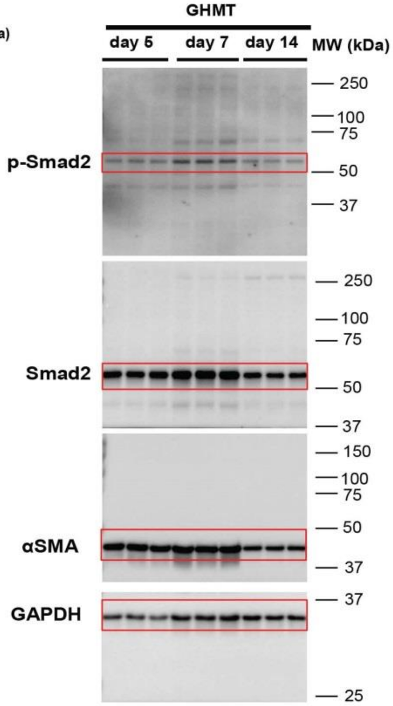

Fig. 3a

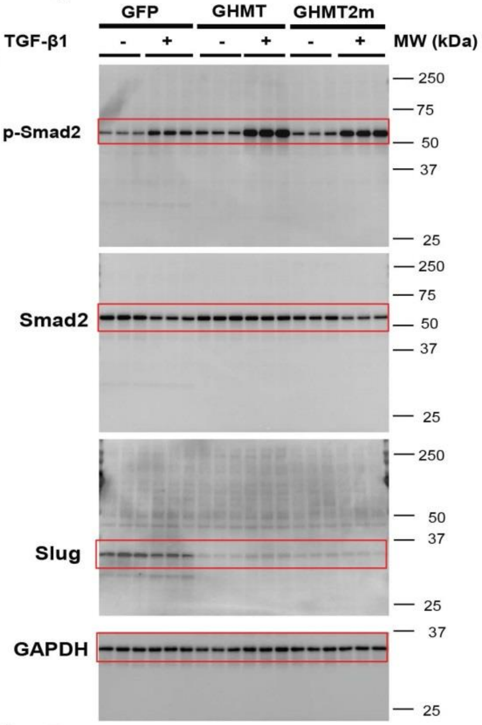

Supplementary Fig. 6b

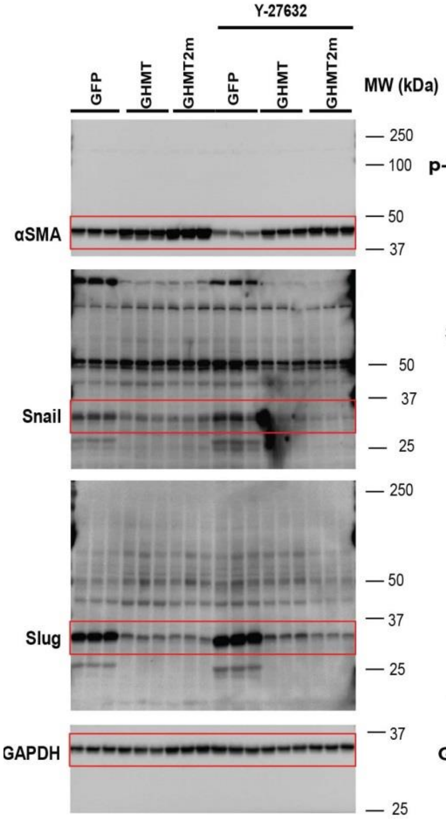

Supplementary Fig. 7a

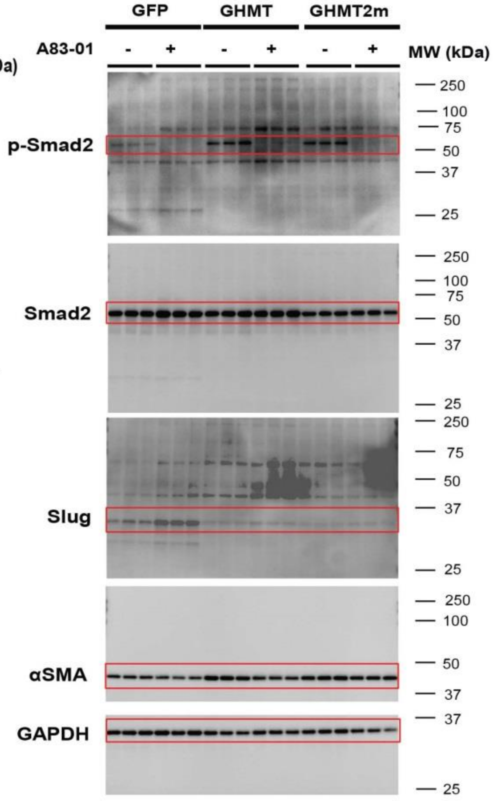

Supplementary Fig. 7g

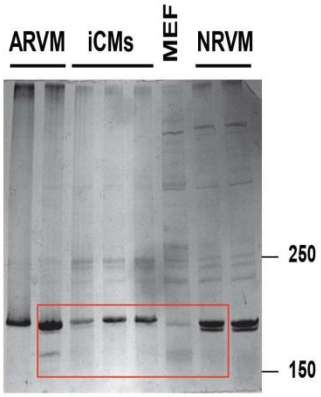

Supplementary Figure 2. Uncropped images of immunoblots displayed in Figs.1b, 1c, 3a, Supplementary Figs. 6b, 7a and 7g. Red boxes highlight lanes used in figures. MW, molecular weight. kDa, kilodaltons.

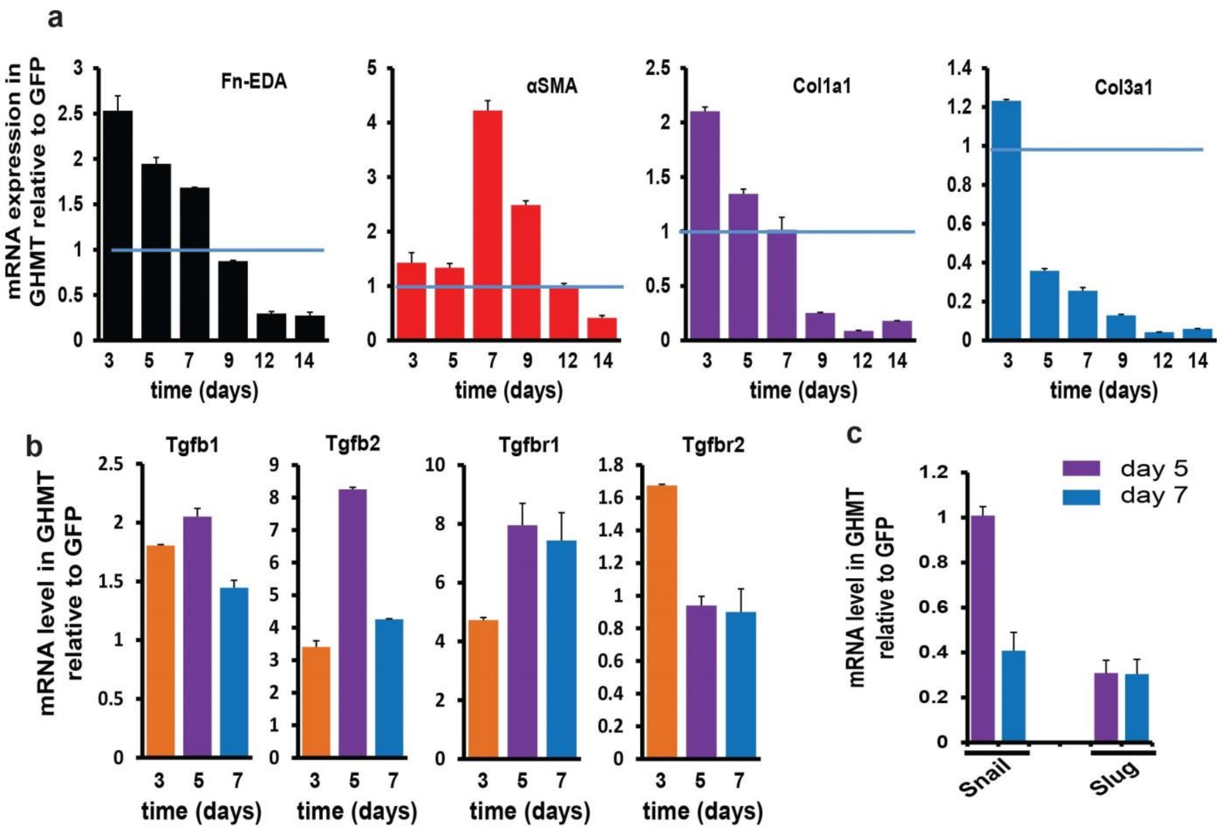

Supplementary Figure 3. Expression of TGF-β signaling components was analyzed at stages of cardiac reprogramming. (**a**) Messenger RNA expression of pro-fibrotic markers, including αSMA, Col1a1, Fn-EDA and Col3a1. Samples were harvested from 3 independent experiments and measured in duplicate. A representative pattern is shown. Data are presented as mean + s.d. (**b**) Time course of expression of the indicated genes in GFP- and GHMT-MEFs was determined by qPCR. A representative pattern from three independent experiments is shown. Data are presented as mean + s.d. (**c**) qPCR analysis for expression of epithelial-to mesenchymal transition (EMT) markers, Snail and Slug, in GFP- and GHMT-MEFs at day 5 and day 7. A representative pattern from three independent experiments is shown. Data are presented as mean + s.d.

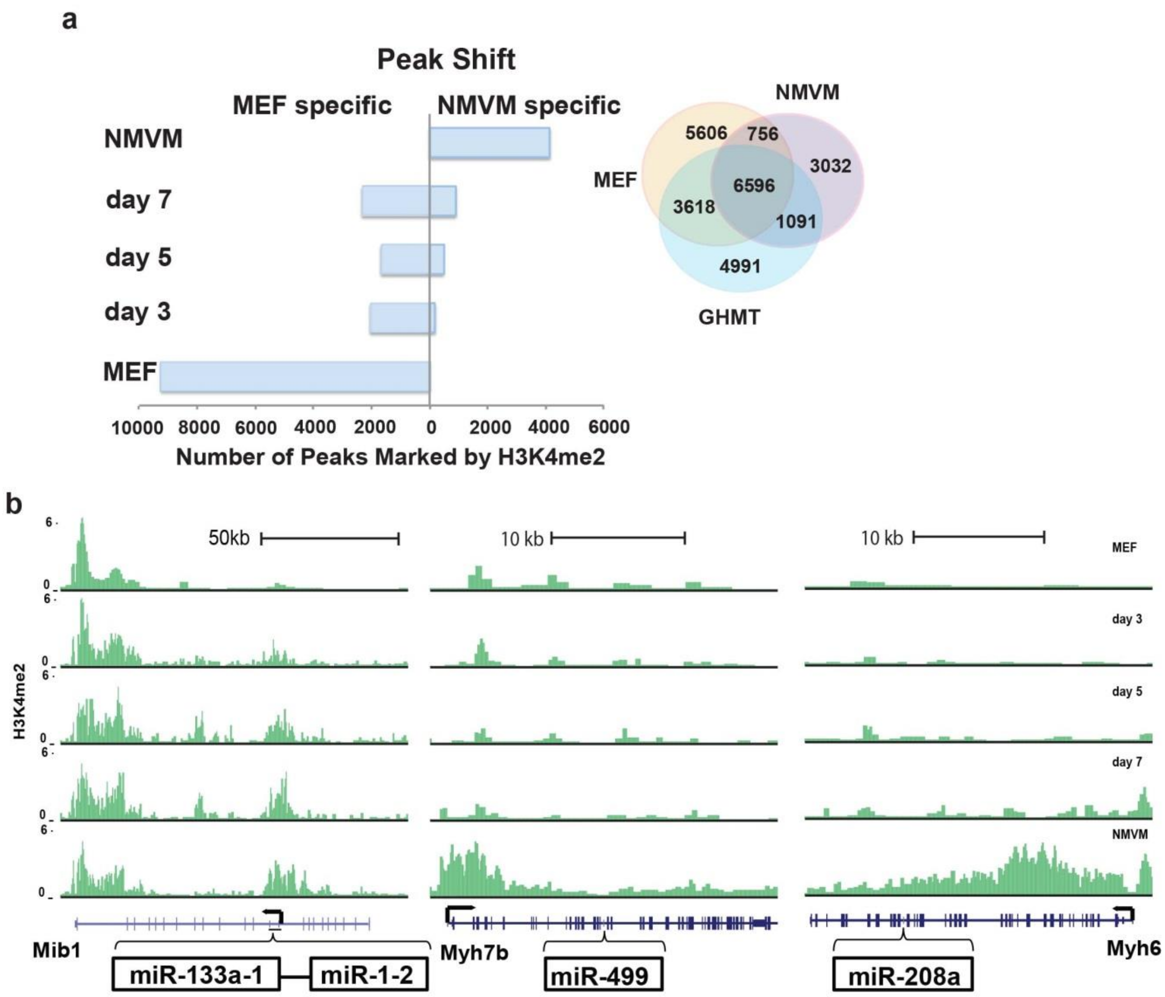

Supplementary Figure 4. Active H3K4me2 marks the cluster of *miR-133a-1* and *miR-1-2* during early induction of reprogramming factors. (a) Number of cardiomyocyte-exclusive or MEF-exclusive H3K4me2 peaks gained or lost during reprogramming. Venn diagram of represented peaks within reprogramming cells at day 7 versus MEFs and primary neonatal mouse ventricular cardiomyocytes (NMVM). (**b**) ChIP-Seq tracks showing a gain of H3K4me2 at the locus of the *miR-133a-1* and *miR-1-2* cluster at the early stages of reprogramming. However, increased H3K4me2 was not detected at *miR-499* and *miR-208* loci at the same time point.

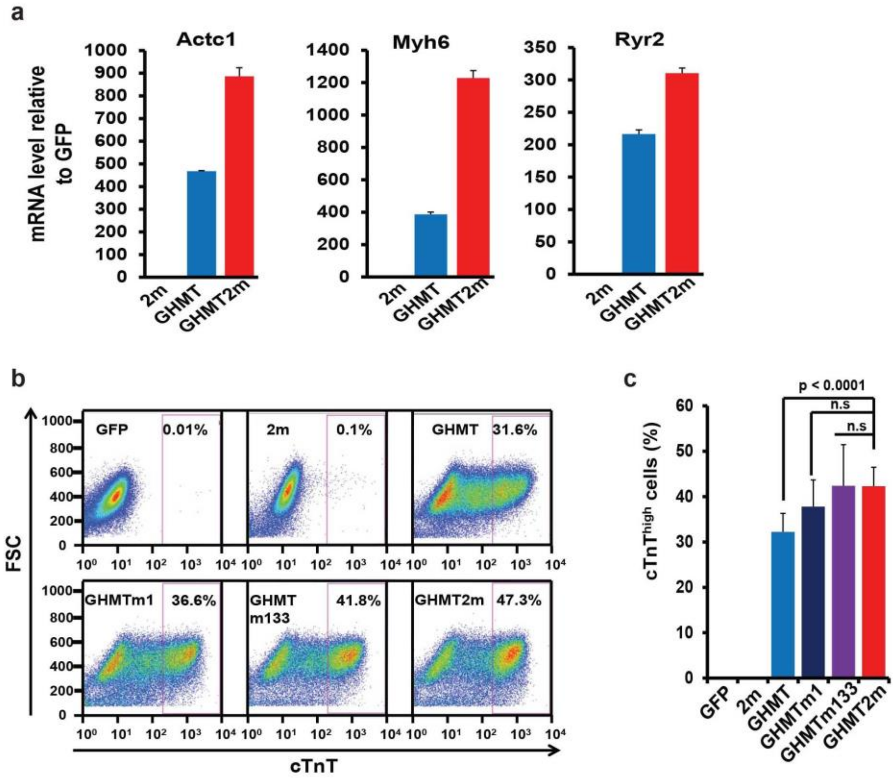

Supplementary Figure 5. miR-1 and miR-133 enhance cardiac reprogramming. (**a**) qPCR analysis of the indicated cardiac genes in MEFs. MEFs were infected with retroviruses carrying the indicated factors and cultured for 7 days. Data are presented as mean + s.d. A representative pattern from two independent experiments is shown. (**b**) Flow cytometry analysis for $cTnT^{+}$ cells at day 7. The number embedded in each plot indicates the percentage of $cTnT^{high}$ cells. 2m (miR-1 + miR-133); GHMTm1 (GHMT + miR-1); GHMTm133 (GHMT + miR-133); GHMT2m (GHMT + miR-1 + miR-133). FSC, forward scatter. (**c**) Quantification of $cTnT^{high}$ cells shown in **b** (n = 4 for GFP, n = 5 for 2m, n = 4 for GHMTm1 and GHMTm133, n = 11 for GHMT and GHMT2m). Data are presented as mean + s.d. $P < 0.0001$ by Student's *t*-test. n.s, not significant.

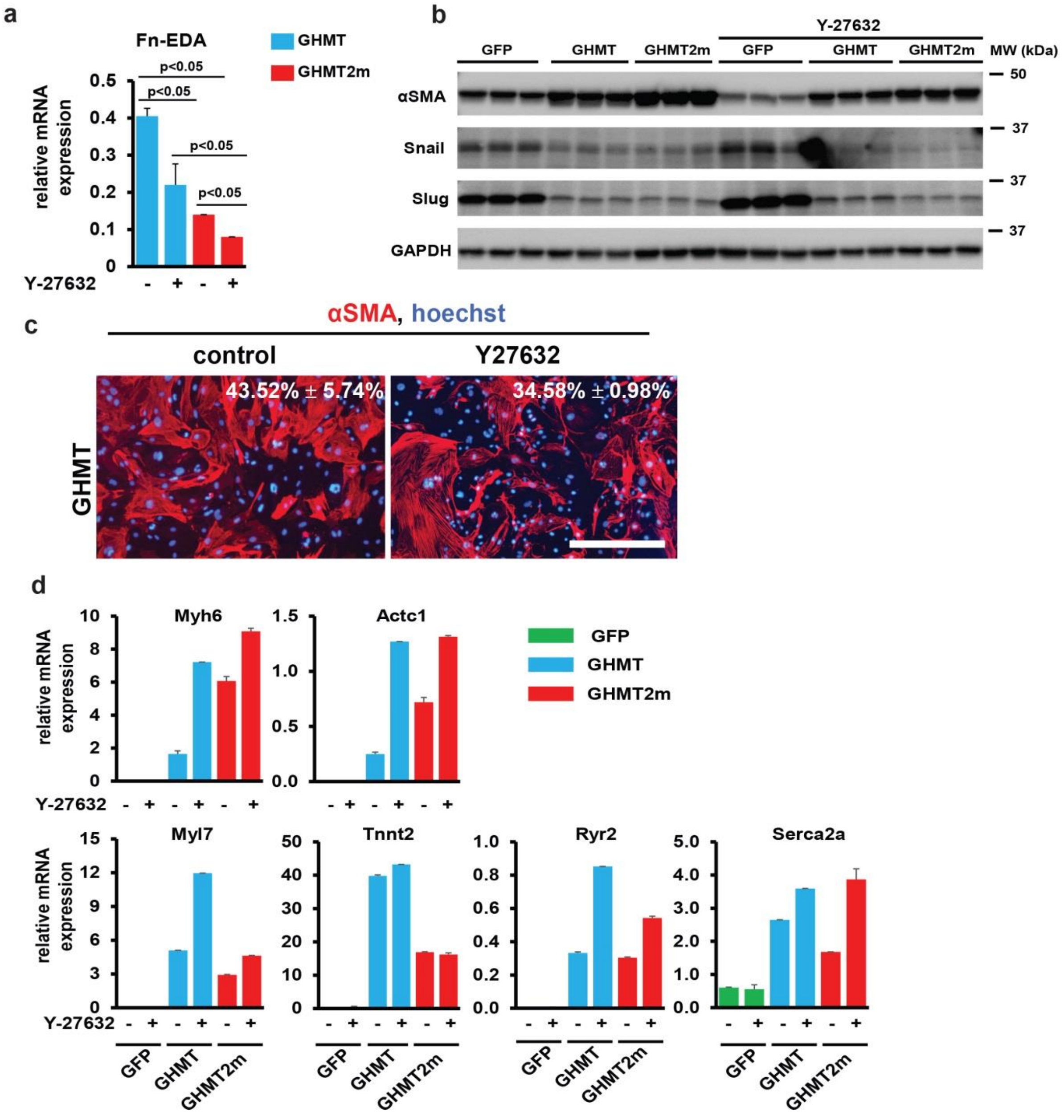

a
Fn-EDA
GHMT
GHMT2m
relative mRNA expression
p<0.05
p<0.05
p<0.05
p<0.05
Y-27632
b
Y-27632
GFP
GHMT
GHMT2m
GFP
GHMT
GHMT2m
MW (kDa)
αSMA
Snail
Slug
GAPDH
50
37
37
37
c
αSMA, hoechst
control
Y27632
GHMT
43.52% ± 5.74%
34.58% ± 0.98%
d
Myh6
Actc1
Myl7
Tnnt2
Ryr2
Serca2a
GFP
GHMT
GHMT2m
relative mRNA expression
Y-27632

Supplementary Figure 6. Y-27632 decreases pro-fibrotic gene expression and enhances cardiac reprogramming. (**a**) qPCR analysis of Fn-EDA in Y-27632 treated reprogramming cells at day 7. Gene expression was normalized to GAPDH. Data are presented as mean + s.d. $P < 0.05$ by Student's *t*-test. N of 4 per group. (**b**) Expression of αSMA, Snail, Slug and GAPDH protein in MEFs overexpressing the indicated factors in the absence or presence of Y-27632 at day 7. Infected MEFs were treated with Y-27632 at 10 μM for 2 days, and then at 30 μM for the other two days. (**c**) Immunostaining of αSMA^+ stress fibers in MEFs overexpressing GHMT in the absence or presence of Y-27632 at day 7. Infected MEFs were treated with Y-27632 for 5 days. The number embedded in each plot indicates the percentage of cells with stress fibers. Scale bar, 400 μm. Data are presented as mean ± s.d. N of 3 per group. (**d**) qPCR analysis for expression of indicated cardiac genes in reprogramming cells at day 7. MEFs infected with retroviruses carrying the indicated factors were treated with Y-27632 or water for 5 days. Gene expression was normalized to GAPDH. Data are presented as mean + s.d. A representative pattern from three independent experiments is shown.

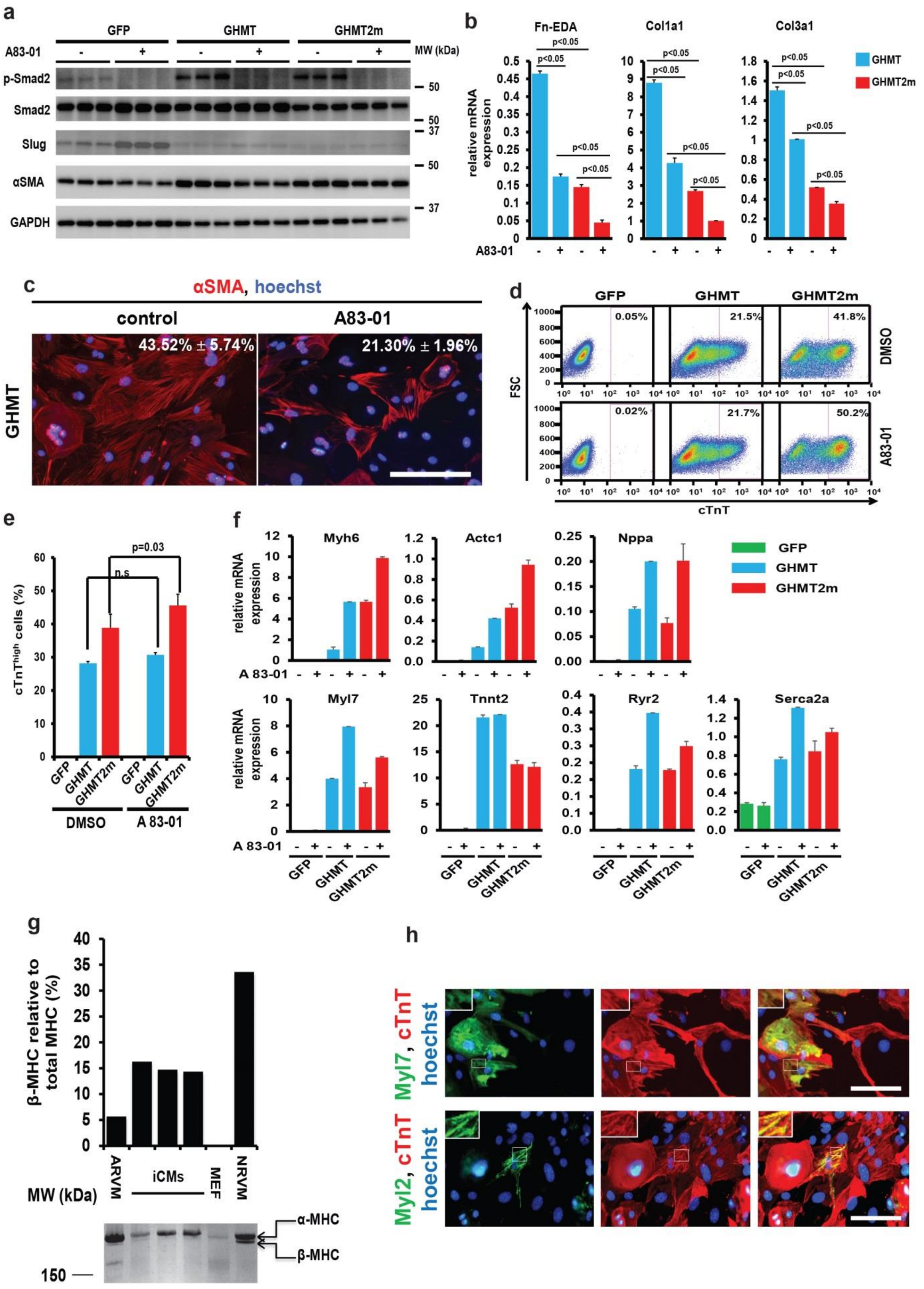
a
GFP
GHMT
GHMT2m
A83-01
MW (kDa)
p-Smad2
Smad2
Slug
αSMA
GAPDH
50
37
b
Fn-EDA
Col1a1
Col3a1
relative mRNA expression
p<0.05
GHMT
GHMT2m
A83-01
c
αSMA, hoechst
control
A83-01
GHMT
43.52% ± 5.74%
21.30% ± 1.96%
d
GFP
GHMT
GHMT2m
0.05%
21.5%
41.8%
0.02%
21.7%
50.2%
DMSO
A83-01
FSC
cTnT
e
p=0.03
n.s
cTnT^high cells (%)
GFP
GHMT
GHMT2m
DMSO
A 83-01
f
Myh6
Actc1
Nppa
Myl7
Tnnt2
Ryr2
Serca2a
GFP
GHMT
GHMT2m
relative mRNA expression
A 83-01
g
β-MHC relative to total MHC (%)
ARVM
iCMs
MEF
NRVM
MW (kDa)
α-MHC
β-MHC
150
h
Myl7, cTnT
hoechst
Myl2, cTnT
hoechst

Supplementary Figure 7. A83-01 decreases pro-fibrotic gene expression and enhances cardiac reprogramming. MEFs were infected with indicated retroviral cocktails, then treated with A83-01 (0.5 µM) or DMSO until samples were harvested. (**a**) Cell lysates at day 7 were immunoblotted with antibodies to phospho-Smad2, Smad2, Slug, αSMA and GAPDH. (**b**) qPCR analysis of indicated gene expression at day 7. Gene expression was normalized to GAPDH. Data are presented as mean + s.d. $P < 0.05$ by Student's *t*-test. N=3. (**c**) Immunostaining of αSMA^{+} stress fibers in MEFs overexpressing GHMT in the absence or presence of A83-01 at day 7. The number embedded in each plot indicates the percentage of cells with stress fibers. Scale bar, 200 µm. Data are presented as mean ± s.d. n = 3. (**d, e**) Flow cytometry analysis for cTnT expression in infected MEFs at day 7. MEFs were infected with the indicated retroviral cocktails, then treated with the TGF-β inhibitor, A83-01 (0.5 µM) or DMSO from day 3 to day 7. The number embedded in each plot indicates the percentage of $cTnT^{high}$ cells in (**d**). FSC, forward scatter. Cells positive for $cTnT^{high}$ were quantified in (**e**). Data are presented as mean + s.d. $P < 0.05$ by Student's *t*-test. n.s, not significant. N=3. (**f**) qPCR analysis for expression of the indicated cardiac genes in reprogramming cells at day 7. MEFs expressing the indicated factors were treated with A83-01 or DMSO for 5 days. Gene expression was normalized to GAPDH. A representative pattern from three independent experiments is shown. Data are presented as mean + s.d. (**g**) Ratio of β-MHC to total MHC (α-MHC + β-MHC) in GHMT2m-cultures (n = 3) treated with A83-01 (0.5 µM) at day 14. A pattern of immunoblotting is shown in the lower panel. Quantification of the ratio is shown in the upper panel. ARVM, adult rat ventricular myocytes; NRVM, neonatal rat ventricular myocytes. (**h)** Representative immunofluorescence images of MEFs treated with GHMT2m plus A83-01 for cTnT (red) and Myl7 or Myl2 (green) by 2 weeks. White boxes are enlarged in insets. Scale bars, 100 µm.

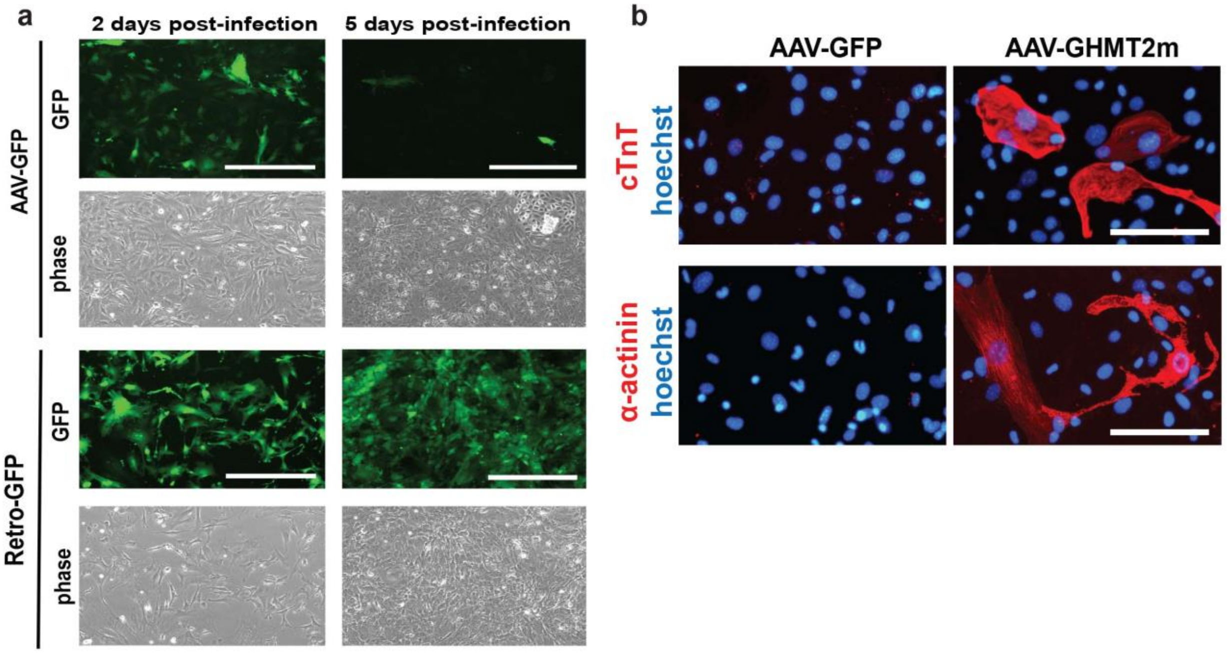

Supplementary Figure 8. GHMT2m delivered by adeno-associated viruses (AAV) reprograms fibroblasts into iCMs. (a) Forced expression of GFP in MEFs delivered by AAV or retrovirus. Scale bars, 400 µm. (**b**) Representative immunofluorescence images of MEFs stained for cTnT (red, upper), and α-actinin (red, lower). MEFs were infected with AAV-GFP or AAV-GHMT2m and treated with A83-01 for 12 days. Scale bars, 100 µm.

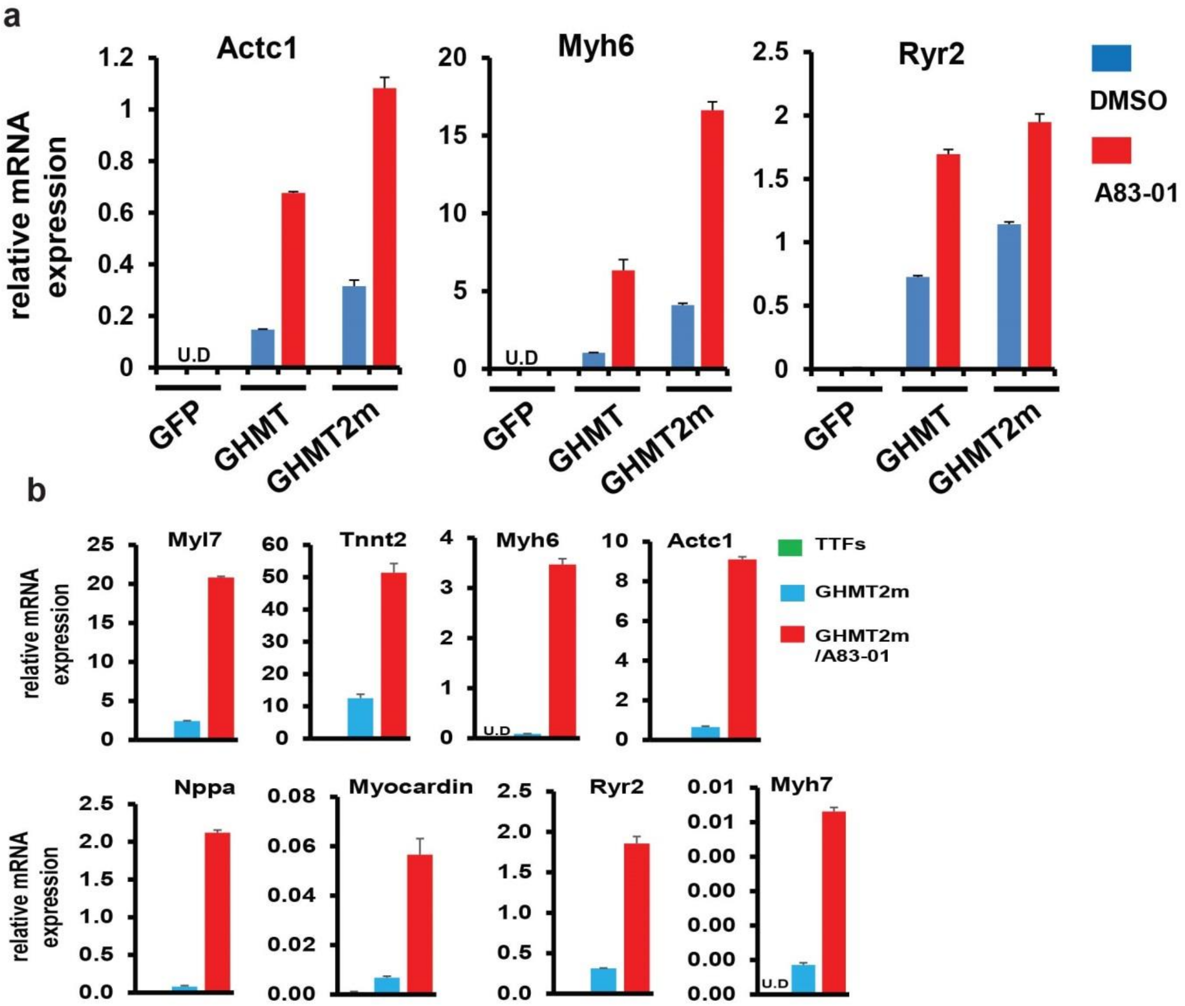

Supplementary Figure 9. Inhibition of pro-fibrotic signaling enhances cardiac reprogramming of adult fibroblasts. (**a, b**) qPCR analysis for expression of the indicated cardiac genes in ACFs (**a**) and ATTFs (**b**). ACFs or ATTFs were infected with retroviruses carrying indicated factors and treated with A83-01 or DMSO for 4 weeks. Gene expression was normalized to GAPDH. Data are presented as mean + s.d. U.D, undetected. A representative pattern from three independent experiments is shown.

Supplementary Table 1. Annotation enrichment analysis of genes regulated in GHMT2m-cultures

Genes		GO Description	Enrichment Score	P-value
Up in GHMT2m	Cardiac Development	contractile fiber part	26.23	4.07E-12
		muscle system process	20.05	1.95E-09
		regulation of heart contraction	19.81	2.48E-09
		myofibril assembly	19.22	4.52E-09
		muscle contraction	17.51	2.49E-08
		voltage-gated cation channel activity	16.33	8.07E-08
		striated muscle contraction	15.91	1.23E-07
		muscle structure development	15.79	1.39E-07
		voltage-gated channel activity	14.13	7.29E-07
		cardiac muscle contraction	13.54	1.31E-06
		calcium channel complex	13.54	1.31E-06
		cardiac muscle cell development	12.29	4.60E-06
		cardiac myofibril assembly	12.22	4.94E-06
		cardiac muscle fiber development	10.04	4.35E-05
		voltage-gated calcium channel complex	9.27	9.40E-05
		voltage-gated potassium channel activity	9.04	1.19E-04
		cardiac muscle tissue morphogenesis	8.91	1.34E-04
		heart development	8.64	1.77E-04
Down in GHMT2m	Fibrotic Events	extracellular region	7.23	7.24E-04
		extracellular matrix	6.94	9.70E-04
		proteinaceous extracellular matrix	6.6	1.36E-03
		extracellular region part	6.07	2.32E-03

RNAs were harvested from GHMT- or GHMT2m-culture at day 7 post-infection. Deep-sequencing was followed by GO analysis. Two hundred and twelve genes were up-regulated ≥ 1.5 folds, and 144 genes were down-regulated ≤ 0.5 folds in GHMT2m-infected cultures. *P*-values were computed by using Partek® Flow® software (Partek).

Supplementary Table 2. Q-PCR primers

Primers	Primer Sequence	References
αSMA .F	TCAGCGCCTCCAGTTCCT	1
αSMA .R	AAAAAAAACCACGAGTAACAAATCAA	
Fn-EDA.F	TTGATTTCTTTCATTGGTCCTGTCTT	2
Fn-EDA.R	AAACAGAAATGACCATTGAAGGTTTG	
Col1a1.F	AGACATGTTCAGCTTTGTGGAC	3
Col1a1.R	GCAGCTGACTTCAGGGATG	
Col3a1.F	ACGTAGATGAATTGGGATGCAG	3
Col3a1.R	GGGTTGGGGCAGTCTAGTG	
Snai2 (Slug).F	CATTGCCTTGTGTCTGCAAG	3
Snai2 (Slug).R	CAGTGAGGGCAAGAGAAAGG	
Snai1.F	GCGGAAGATCTTCAACTGCAAATATTGTAAC	4
Snai1.R	GCAGTGGGAGCAGGAGAATGGCTTCTCAC	
GAPDH.F	GCAGTGGCAAAGTGGAGATTG	
GAPDH.R	GGAGATGATGACCCTTTTGGCTCC	
Tgfb1.F	TGGAGCAAC ATGTGGAACTC	5
Tgfb1.R	GTCAGCAGCCGGTTACCA	
Tgfb2.F	AGGAGTGGCTTCACCACAAAGACA	6
Tgfb2.R	ATTAGACGGCACGAAGGTACAGCA	
Tgfbr1.F	CATTCACCACCGTGTGCCAAATGA	6
Tgfbr1.R	ACCTGATCCAGACCCTGATGTTGT	
Tgfbr2.F	TCCCAAGTCGGATGTGGAAATGGA	7
Tgfbr2.R	TCGCTGGCCATGACATCACTGTTA	
Nppa.F	TTCTTCCTCGTCTTGGCCTTT	8
Nppa.R	GACCTCATCTTCTACCGGCATCT	
Myh6.F	GCCCAGTACCTCCGAAAGTC	9 (ID: 6754774a1)

Myh6.R	GCCTTAACATACTCCTCCTTGTC	
TnnT2.F	CAGAGGAGGCCAACGTAGAAG	9(ID: 6755843a1)
TnnT2.R	CTCCATCGGGGATCTTGGGT	
Myl7.F	GGCACAACGTGGCTCTTCTAA	9 (ID:12584966a1)
Myl7.R	TGCAGATGATCCCATCCCTGT	
Actc1.F	GTATGCTTCTGGAAGAACTACA	
Actc1.R	GCAGTGGTGACAAAGGAGTAC	
Myocardin.F	CTGTGTGGAGTCCTCAGGTCAAACC	10
Myocardin.R	GATGTGCTGCGGGCTCTTCAG	
SERCA2a.F	GGCCAGATCGCGCTACA	11
SERCA2a.R	GGGCCAATTAGAGAGCAGGTTT	

Supplementary References

1. Henderson, N.C. et al. Galectin-3 regulates myofibroblast activation and hepatic fibrosis. *Proc. Natl. Acad. Sci. U. S. A.* 103, 5060-5 (2006).
2. Baelde, H.J. et al. Alternatively spliced isoforms of fibronectin in immune-mediated glomerulosclerosis: the role of TGFbeta and IL-4. *J. Pathol.* 204, 248-57 (2004).
3. Smith, C.L., Baek, S.T., Sung, C.Y., & Tallquist, M.D. Epicardial-derived cell epithelial-to-mesenchymal transition and fate specification require PDGF receptor signaling. *Circ. Res.* 108, e15-26 (2011).
4. Luo, D., Wang, J., Li, J., & Post, M. Mouse snail is a target gene for HIF. *Molecular cancer research : MCR.* 9, 234-45 (2011).

5. Imai, K. et al. Inhibition of dendritic cell migration by transforming growth factor-beta1 increases tumor-draining lymph node metastasis. *J. Exp. Clin. Cancer Res.* 31, 3 (2012).
6. Zhang, M. et al. Myocardial remodeling is controlled by myocyte-targeted gene regulation of phosphodiesterase type 5. *J. Am. Coll. Cardiol.* 56, 2021-30 (2010).
7. Gore, A.J., Philips, D.P., Miller, W.L., & Bernard, D.J. Differential regulation of follicle stimulating hormone by activin A and TGFB1 in murine gonadotropes. *Reprod. Biol Endocrinol.* 3:73 (2005).
8. Matsumoto, E. et al. Angiotensin II-induced cardiac hypertrophy and fibrosis are promoted in mice lacking Fgf16*. Genes to Cells* 18, 544-53 (2013).
9. http://pga.mgh.harvard.edu/primerbank/(2015)
10. Zhao, R. et al. GATA6 is essential for embryonic development of the liver but dispensable for early heart formation. *Mol. Cell Biol.* 25, 2622-31 (2005).
11. Ferguson, B.S. et al. Signal-dependent repression of DUSP5 by class I HDACs controls nuclear ERK activity and cardiomyocyte hypertrophy. *Proc. Natl. Acad. Sci. U. S. A.* 110, 9806-11 (2013).
